# Zirconium‐Assisted Activation of Palladium To Boost Syngas Production by Methane Dry Reforming

**DOI:** 10.1002/anie.201807463

**Published:** 2018-09-17

**Authors:** Norbert Köpfle, Thomas Götsch, Matthias Grünbacher, Emilia A. Carbonio, Michael Hävecker, Axel Knop‐Gericke, Lukas Schlicker, Andrew Doran, Delf Kober, Aleksander Gurlo, Simon Penner, Bernhard Klötzer

**Affiliations:** ^1^ Institute of Physical Chemistry University of Innsbruck Innrain 52 c 6020 Innsbruck Austria; ^2^ Department of Inorganic Chemistry Fritz-Haber-Institute of the Max-Planck-Society Faradayweg 4–6 14195 Berlin Germany; ^3^ Helmholtz-Zentrum Berlin für Materialien und Energie GmbH BESSY II Albert-Einstein-Straße 15 12489 Berlin Germany; ^4^ Fachgebiet Keramische Werkstoffe Institut für Werkstoffwissenschaften und -technologien Technische Universität Berlin Hardenbergstr. 40 10623 Berlin Germany; ^5^ Advanced Light Source, Beamline 12.2.2. Lawrence Berkeley National Laboratory Berkeley CA 94720 USA

**Keywords:** CO_2_ activation, heterogeneous catalysis, methane dry reforming, photoelectron spectroscopy, X-ray diffraction

## Abstract

C‐saturated Pd^0^ nanoparticles with an extended phase boundary to ZrO_2_ evolve from a Pd^0^Zr^0^ precatalyst under CH_4_ dry reforming conditions. This highly active catalyst state fosters bifunctional action: CO_2_ is efficiently activated at oxidic phase boundary sites and Pd_*x*_C provides fast supply of C‐atoms toward the latter.

Dry reforming of methane (DRM) allows to convert climate‐harming methane and carbon dioxide into useful syngas, implying its practical use for greenhouse gas abatement and CO_2_ utilization.^1^ Ideally, a stoichiometry of CH_4_+CO_2_→2 H_2_+2 CO is obtained. The resulting 1:1 syngas ratio is rather useful for subsequent carbonylation or hydroformylation processes.^2^ If the H_2_ yield can be enhanced, for example, by using membrane reactors, also the synthesis of renewable fuels may become attractive.^3^ Practical problems are associated with loss of H_2_ selectivity owing to the water‐gas‐shift equilibrium, especially at elevated pressures^4^ and with irreversible coking phenomena, especially on the less‐costly Ni‐based catalysts.^5^, ^6^, ^7^, ^8^ Alternatively, a series of highly active noble metal catalysts deserve attention from a fundamental viewpoint, due to superior coking properties.^6^, ^7^, ^9^, ^10^ Empirical attempts to impart this coking resistance to nickel without sacrificing activity led to a series of promising bimetallic catalysts, with NiPd/ZrO_2_ representing one of the best.^11^ Yet, the mechanistic effects of noble metal alloying of Ni are not clear. Besides potential ensemble and electronic structure effects at the bimetallic surface^12^, ^13^ different levels of metal oxide bifunctionality have been proposed, depending on the intrinsic activity of the respective pure metal component.^10^ Pure Ni surfaces are in principle capable of activating both CH_4_ and CO_2_,^13^, ^14^, ^15^ and thus a minor co‐catalytic role of the support can be anticipated. In contrast, a high degree of bifunctionality can be expected, if a very good CH_4_ activator such as Pd exhibits comparatively poor CO_2_ activation properties.^10^, ^15^, ^16^ Consequently, only the promotion of CO_2_ activation and subsequent CO product formation at both active and abundant oxide support: Pd interface sites can establish high DRM activity. In such cases, empirical development of catalyst preparation and catalyst activation must logically aim in a particularly large (bi)metal interface to an oxidic support with good CO_2_ activation kinetics. Critical parameters determining the latter encompass surface reducibility, basicity, reactivity of oxygen vacancies toward CO_2_, and so on.^17^

Metal‐wise, both fast supply of reactive carbon toward and high abundance at the metal oxide phase boundary (abbreviated as PB in the following) is mandatory. Pd exhibits excellent subsurface and bulk carbon diffusion and storage properties, which is not only important for a variety of catalytic applications^18^ but also for graphene and carbon nanotube growth^18^, ^19^ and novel (electro)catalytic applications of graphene‐covered Pd nanostructures.^20^ Thus, the experimental and theoretical investigation of carbon resegregation and reactivity on Pd is a topic of general interest.^21^, ^22^

Owing to their structural heterogeneity and the limited applicability of surface‐sensitive in situ spectroscopy techniques such as AP‐XPS, technical powder catalysts usually do not allow to extract unambiguous evidence for the catalytic role of the PB. The use of conductive (bi)metallic substrates, on which a thin active layer forms under reaction conditions by oxidative segregation, both allows the circumvention of conductivity problems and provides a quasi‐2D region of spectral observation. Thus, both a surface and bulk bimetallic PdZr model catalyst approach toward active PB sites was employed. The initial and DRM‐induced states of these model systems are represented in the Supporting Information, Figure S1. A CVD‐prepared Zr^0^ subsurface alloy state on Pd foil showed oxidative segregation of ZrO_*x*_H_*y*_ domains at the Pd surface under DRM conditions, thus forming an inverse ZrO_*x*_H_*y*_ island on bulk Pd model catalyst, which showed to be somewhat more active than its individual components ZrO_2_ and Pd.^23^ Alternatively, the in situ activation of bulk intermetallic precursors is a particularly efficient way to generate a large amount of PB sites between (bi)metallic and oxidic nanoparticles, as shown in the lower part of the Supporting Information, Figure S1.^24^ Both approaches were followed in this work, using a combination of synchrotron‐based XRD and XPS analysis under DRM conditions to characterize both the bulk‐related phase changes and the active surface/interface state in situ.

Details of sample preparation of the bulk‐intermetallic and sub‐surface alloy Pd^0^Zr^0^ samples are given in the Experimental Section of the Supporting Information and in previous reports.^23^, ^25^ The partially oxidized pre‐reaction surface state on top of the bulk‐intermetallic substrate was characterised by XPS (Supporting Information, Figure S2). To characterize the bulk composition of the precatalyst versus the DRM operando state, in situ XRD spectra were taken before and during reaction. The corresponding effects are shown in Figure 1. The relevant 2*θ* range of the integrated data can be accordingly seen in (a). Half of the recorded detector image, including the phase assignment, is displayed in (b).

**Figure 1:**
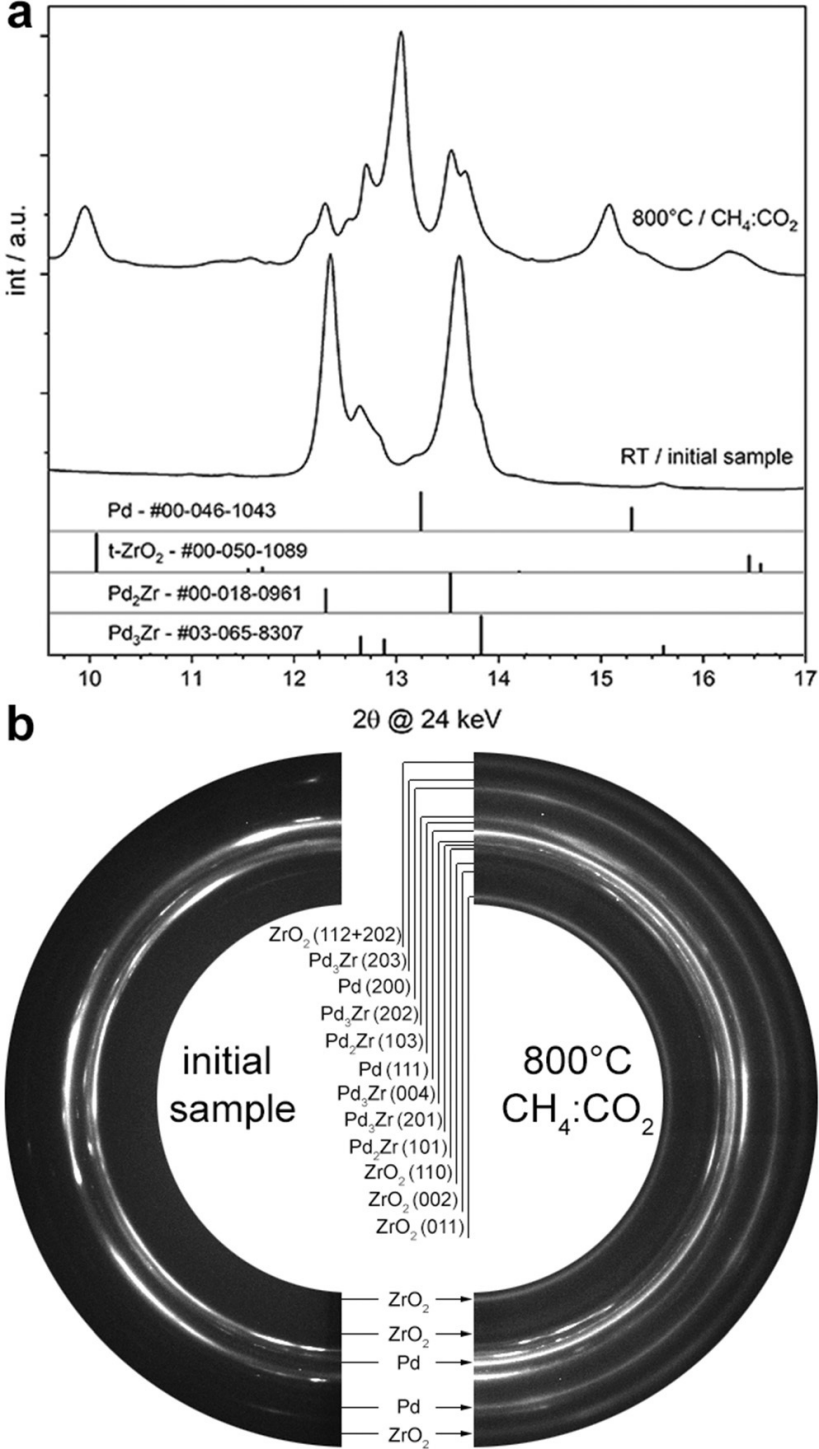
In situ XRD of the bulk‐intermetallic Pd_*x*_Zr_*y*_ catalyst at ca. 20 °C and during DRM at 800 °C. A gas flow of 2 mL min^−1^ of CH_4_/CO_2_ (ratio 1:1) at ambient pressure with a heating rate of 20 K min^−1^ was applied. Initial state at room temperature: a), bottom spectrum and b), left side; in situ DRM state: a), top spectrum and b), right side.

The initial sample contains Pd_2_Zr and Pd_3_Zr and is coarse crystalline, as can be derived from the spotty, that is, not completely continuous, rings. Under DRM conditions, metallic and crystalline Pd is formed, as well as tetragonal ZrO_2_. At the same time, the fraction of Pd_2_Zr and Pd_3_Zr in the phase mixture decreases, inferred from the significantly lower intensities of the associated reflexes compared to the initial sample. Both newly formed phases, namely Pd and tetragonal (t‐)ZrO_2_, appear as completely continuous rings in the detector image and therefore, have a fine or nanocrystalline morphology. Both the crystallite size and the lattice constant of Pd^0^ can be estimated via Rietveld analysis, yielding a value of 3.914 Å and a mean crystallite size of about 7.5 nm during DRM at 800 °C. As will be shown in the context of Figure 3, in situ XPS clearly revealed a C_bulk_ loaded carbidic state of Pd^0^ under DRM conditions. Accordingly, a pronounced lowering of the Pd^0^ lattice parameter is observed (Supporting Information, Figure S3) that is due to O_2_‐induced C_bulk_ depletion just at the onset of partial Pd oxidation (3.898 Å, 10.3 nm mean crystallite size).

Quantitative temperature‐programmed DRM reaction studies on both the subsurface and bulk intermetallic precatalysts were performed in our UHV‐compatible recirculating batch reactor (for details, see the Supporting Information, Experimental Section), and compared to the activities of clean Pd metal foil and a fully ZrO_2_ covered Zr foil. The bulk‐intermetallic rate data are shown in Figure 2, revealing a strong, 33‐fold DRM rate promotion relative to a single‐phase ZrO_2_ oxide film. Pure Pd foil shows hardly any measurable activity. At about 860 K the CO formation rate starts to increase exponentially with the temperature and exhibits a maximum upon reaching the final temperature of 1073 K. The subsequent isothermal rate decrease is caused by progressive reactant consumption (CH_4_ and CO_2_ conversion after 45 min: ca. 96 %). We note that the CO:H_2_ product ratio was close to 1:1 and accordingly, the ratio of the CO_2_ consumption vs. CO formation rates close to 1:2.

**Figure 2:**
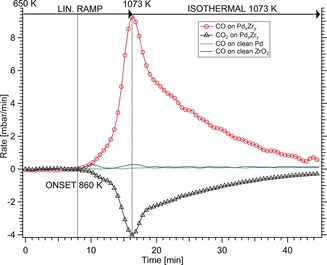
Temperature‐programmed DRM rate profile on the Pd_*x*_Zr_*y*_ bulk‐intermetallic precatalyst, plotted versus the rates measured on clean Pd foil and clean ZrO_2_. Reaction conditions: 50 mbar CH_4_, 50 mbar CO_2_, 977 mbar He; linear temperature ramp (25 K min^−1^) up to 1073 K, followed by isothermal reaction for 30 min.

The complementary DRM rate data on the PdZr subsurface alloy precatalyst are plotted in the Supporting Information, Figure S4, showing only slightly higher average rates than on pure ZrO_2_.

The DRM activity is effectively not scalable with the Pd^0^/Zr(ox) interface sites forming in situ under DRM conditions on bulk Pd^0^ (for details, see the Supporting Information, Figure S4). Rather, it is synergistically boosted by the combination of an extended PB with nanoparticulate Pd^0^. The XRD results of Figure 1 show that the PB forms in situ by oxidative t‐ZrO_2_ segregation from Pd^0^Zr^0^ in the DRM gas phase, and that this process provides Pd^0^ in a nanodispersed form. The related C 1s core‐level spectra obtained by AP‐XPS on the bulk PdZr precatalyst are shown in Figure 3. They provide clear indications of considerable C_bulk_ concentrations within Pd, but only in the presence of CH_4_ gas. The C 1s component at a BE of 283.0 eV (upper panel, green), as well as the Pd 3d component at 335.6 eV (lower panel, green) correspond well to literature‐reported values of 335.6 eV and 282.9 eV for Pd modified by bulk‐dissolved carbidic/interstitial C^22^, ^26^ and are obviously linked to ongoing C supply via the gas phase. The bulk character of this species is supported by its response to a change of the photon energy/C 1s kinetic energy, as shown in the Supporting Information, Figure S5. Upon extending the probe depth, its relative intensity to the surface‐graphitic component increases. As soon as the CH_4_ supply is switched off, the C_bulk_ signal disappears immediately, whereas the C_graphite_ component decreases at a slower rate. Obviously, C_bulk_ is much more reactive than C_graphite_ with respect to the carbon clean‐off reaction in pure CO_2_. The C_graphite_ intensity decreases even more slowly below the level shown in Figure 3 for pure CO_2_, which can be explained by a certain amount of even less reactive carbon resulting from ambient exposure prior to catalysis (for details, see the Supporting Information, Figure S6).

**Figure 3:**
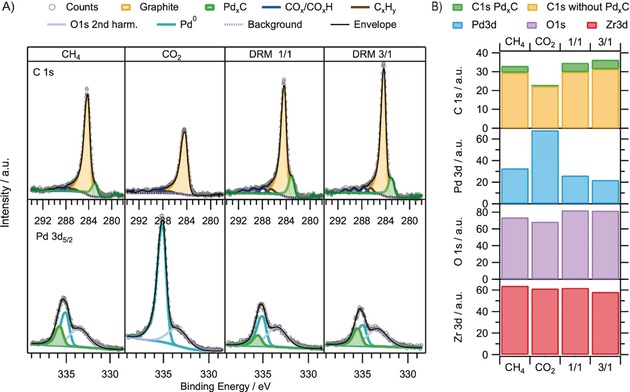
C 1s and Pd 3d_5/2_ AP‐XPS spectra recorded at a common kinetic energy of 400 eV in situ during DRM at ca. 700 °C, starting from the bulk Pd_*x*_Zr_*y*_ precursor. Applied gas pressures from left to right: 0.3 mbar CH_4_, 0.3 mbar CO_2_, DRM 1:1=0.15 mbar CH_4_+0.15 mbar CO_2_, DRM 3:1=0.225 mbar CH_4_+0.125 mbar CO_2_. A) Corresponding C 1s and Pd 3d_5/2_ peak deconvolution; B) XPS intensity bar graph of the integrated C 1s, Pd 3d, O 1s, and Zr 3d regions.

The related C 1s and Pd 3d spectra on the initial Pd/Zr^0^ subsurface alloy exhibit no such carbidic/interstitial C_bulk_ component under otherwise identical DRM conditions(Supporting Information, Figure S7). The fundamental difference between the two model systems regards the dimensions of the respective Pd bulk. In case of the initial subsurface alloy, the in situ formed ZrO_*x*_H_*y*_ domains reside on a practically infinite Pd bulk, which will permanently lose C_bulk_ by diffusion into deeper regions, and thus will require a very long time to reach C‐saturation. In contrast, the bulk intermetallic decomposes toward Pd nanoparticles in close contact to t‐ZrO_2_ domains. The reduced Pd bulk dimensions obviously facilitate/accelerate the accumulation of interstitially dissolved C_bulk_.

As a consequence, carbon resegregation from sufficiently C‐supersaturated Pd regions will enhance the surface‐near C_ads_ concentration and, in due course, the nucleation/growth of graphene/graphite domains.^27^ In the same turn, an enhanced rate of C bulk and surface diffusion toward the PB must result. From the combination of the extended PB and the reduced Pd metal dimensions, the much higher reaction rates on the bulk intermetallic precursor can be rationalized. Still, the exact spatial distribution of the ZrO_2_, graphitic, and Pd_*x*_C domains remains to be clarified under analogous DRM conditions, for example by using in situ HRTEM techniques.

On both model systems, the C_graphite_ species exhibit a dynamic and reversible response both to changes of the gas phase composition and of the temperature. On the bulk intermetallic system, the C_graphite_ species reacted much more slowly than the C_bulk_ species. We observed on both model systems that the C_graphite_ intensities (and on the initial bulk intermetallic also the C_bulk_ intensities) reached a steady‐state value after establishing constant partial pressure and temperature conditions. These steady state values are depicted on the right sides of Figure 3 (bulk intermetallic precursor) and the Supporting Information, Figure S7 (subsurface Zr^0^), together with the related final Zr 3d, O 1s, and Pd 3d intensities. From the relative intensity changes in Figure 3, a preferential accumulation of carbon on the Pd particles can be deduced, as increasing methane partial pressures lead to a dominant screening of the Pd 3d signal. In turn, the oxide surface‐related Zr 3d and O 1s intensities rather show a weak or even opposite response. This behavior is in strong contrast to the intensity trends observed on the initial subsurface‐Zr^0^ system, where preferential screening of the Zr 3d and O 1s signals is observed upon C accumulation. Again we suggest that, despite of continuous C supply via CH_4_(g) to Pd, the quasi‐infinite dimensions of the Pd foil allow for permanent C loss to deeper bulk regions, causing little C‐supersaturation in surface‐near regions and thus no or heavily delayed graphite nucleation/growth. Especially under the influence of the highly intense and ionizing synchrotron X‐ray beam, C accumulation on top of the oxidatively segregated ZrO_*x*_ domains is thus preferred. Moreover, C‐diffusion through the surface‐ZrO_*x*_ domains is expected to be hardly possible or at least kinetically strongly retarded. The combination of these effects can eventually explain the observed preferential C‐accumulation on the oxidic domains. This carbon can be reacted off quantitatively in pure CO_2_, likely because of intrinsically close vicinity to the active PB.

By increasing the temperature, the clean‐off reaction rate at the PB of the initial PdZr bulk model is enhanced, and above 700 °C a lowering of the steady‐state C 1s signal results, as shown in the Supporting Information, Figure S8. This is likely due to a changed balance of graphitic C formation and redissolution rates. This effect could also imply a possible mechanistic explanation for the intermediate temperature coking region^28^ of DRM, which can be interpreted by a shift of the Boudouard equilibrium toward carbon. Assuming a faster increase of the C clean‐off rate at the PB with temperature in comparison to the net rate of partially reversible graphene/graphite accumulation, the inverse Boudouard process C+CO_2_→2 CO at the PB will simply overtake the net C_graphite_ deposition rate at sufficiently high temperatures.

In conclusion, the surface‐near regions of the bulk PdZr pre‐catalyst are oxidatively decomposed under realistic DRM conditions and the resulting Pd^0^ nanoparticles provide an appropriate near‐surface carbon loading at the resulting Pd^0^/t‐ZrO_2_ interface at about 700 °C, as depicted in Scheme 1. This, in turn, creates optimized conditions for bifunctional catalyst operation: Pd^0^ regions favor fast supply of reactive C‐atoms toward the phase boundary, whereas Zr(ox) sites^10^, ^16^ assist in CO_2_ activation and the transfer of CO_2_‐derived oxygen to the latter, thus providing optimum conditions for high CO‐activity.

**Scheme 1:**
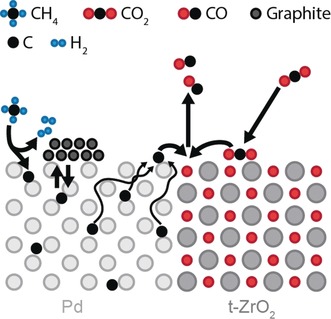
Proposed DRM mechanism leading to enhanced CO formation at the Pd/t‐ZrO_2_ interface.

Empirical attempts to control coking of, for example, Ni by specifically active supports such as CeZrO_*x*_ 5, 29 seem to make use of this principle. Faster C‐depletion of the metallic component via an accelerated phase boundary reaction can directly lower the C concentration of the metal particles and thus initially disfavor nucleation and growth of graphite‐type C‐species, but also enhance the relative amount of redissolution of the latter in the metal as C_bulk_ under stationary reaction conditions. This scenario provides a solid basis for directional promotion of microkinetic steps leading both to enhanced activity and improved control of carbon chemistry during DRM. Important implications for knowledge‐based DRM catalyst synthesis, at least if C‐dissolving metals such as Ni and Pd are involved, are: 1) optimization of phase boundary dimensions and CO_2_ activation properties of the support; 2) adjustment of (bi)metal particle size to achieve C_bulk_ depletion even in metallic regions with the largest distance to the phase boundary; 3) use of (bi)metallic catalysts with suppressed nucleation‐ and growth kinetics of graphite‐type C‐species, at least within the DRM temperature range; and 4) high abundance and reactivity of interfacial C_ads_ species, achievable via optimized CH_4_ adsorption/sticking at the metallic surface and fast bulk/surface diffusion to not too strongly C‐binding sites at or close to the PB. As shown in this work, the use of intermetallic precursors such as Pd_*x*_Zr_*y*_ is one way to match these criteria.

As a perspective for future research, the detailed reaction mechanism of CO_2_ splitting toward reactive oxygen‐, carbonate‐ and/or formate‐type species^17^, ^30^ at the PB remains to be clarified. Distinct oxygenate intermediates were observed in this study by AP‐XPS (Figure 3; Supporting Information, Figures S2, S5, S7), which appear to reside at the PB or the oxidic surface, and which exhibit a partially reversible dynamic response to gas‐phase‐ and temperature changes. In the context of oxygenated C1 species, support acidity vs. basicity has been proposed to influence their specific type and distribution at the PB.^31^ In principle, CO_2_ splitting via reactive vacancies at the PB can proceed directly or via intermediate vacancy‐bonded oxygenates.^32^, ^33^ Clearly, the observed oxygenate C 1s species deserve future experimental and theoretical attention, as the poor data quality and ambiguous background correction in the region above about 286 eV did not allow the assignment of any of the species safely or the distinguishing of in situ gas‐phase induced intensity trends from other background effects without doubt.

(Bi)metal‐wise, further fundamental work should aim in directional promotion of C_ads_ reactivity via electronic modulation of, for example, C bond strength^34^ in combination with lowered barriers for C_bulk_ diffusion and redissolution of C_graphite_, to enhance the PB‐abundance of reactive C atoms.

## Conflict of interest

The authors declare no conflict of interest.

## Supporting information

As a service to our authors and readers, this journal provides supporting information supplied by the authors. Such materials are peer reviewed and may be re‐organized for online delivery, but are not copy‐edited or typeset. Technical support issues arising from supporting information (other than missing files) should be addressed to the authors.

Supplementary
