# Supplementary material for: Zirconium‐Assisted Activation of Palladium To Boost Syngas Production by Methane Dry Reforming
Source: Angew Chem Int Ed Engl. 2018 Sep 17;57(44):14613–8. doi: 10.1002/anie.201807463 (PMC6221108; doi:10.1002/anie.201807463)
Supplement: Supplementary file 1 — Supplementary [file ANIE-57-14613-s001.pdf]

Angewandte Chemie
Eine Zeitschrift der Gesellschaft Deutscher Chemiker

Supporting Information

Zirconium-Assisted Activation of Palladium To Boost Syngas Production by Methane Dry Reforming

*Norbert Köpfle, Thomas Götsch, Matthias Grünbacher, Emilia A. Carbonio, Michael Hävecker, Axel Knop-Gericke, Lukas Schlicker, Andrew Doran, Delf Kober, Aleksander Gurlo, Simon Penner, and Bernhard Klötzer**

anie_201807463_sm_miscellaneous_information.pdf

Supporting Information

Experimental Section

Details of sample preparation:

The preparation of the bulk-intermetallic Pd_xZr_y sample was realized by resistive heating of a stack of alternating small pieces of pure, clean Pd and Zr under high vacuum conditions (1×10^{-7}mbar) at a nominal ratio = 2:1 in a Ta crucible. When the sample was heated slightly above the melting point of Pd (1828 K), a spontaneous exothermic reaction between Pd and Zr took place, leading to an intermetallic Pd-Zr melt. The heating was turned off immediately and the melt recrystallized to form the Pd-Zr bulk phase mix characterised in Figure 1 of the main paper.

As reference catalyst and substrate for PdZr sub-surface alloy formation, ultra-clean Pd foil (GoodFellow, purity 99,95%, 0.1 mm thick, size ~7 cm^2) was used. Surface preparation and characterization involved the usual cycles of Ar sputtering and thermal annealing (6.0 x 10^{-5} mbar Ar, 2 keV, 1 µA sample current; T_{anneal} = 700°C), till XPS and AES spectra without evidence for impurity traces were obtained.

For the Pd/Zr^0 sub-surface alloy preparation, we used Zirconium (IV) tert-butoxide ($Zr(O\text{-}t\text{-}C_4H_9)_4$ (ZTB, Sigma Aldrich, purity: 99.999 %) as the CVD precursor molecule, which was dosed via a leak valve onto the Pd foil substrate in the ultrahigh vacuum chamber of Setup 1 (described below). After an exposure at a pressure of 5,0 x 10^{-6} mbar for 4 min at 400°C, corresponding to 1,2 x 10^{-3} mbar*sec, an adsorbate coverage of ~0.5 ML was calculated using the post-deposition XPS data. Details of the XPS quantification can be derived from [1]. The resulting $Zr^{+4}O_xH_y$ state on top of Pd^0 was then thermally annealed at ~430 °C under very good UHV conditions. Only if the base pressure of the system was kept below $5x10^{-10}$ mbar during thermal annealing, 100% conversion to the Zr^0 subsurface alloy state was achieved, which represents a strong improvement of the preparation routine relative to the partially oxidised precatalyst state described in [2]. The 100% subsurface alloy state was proven via the combination of low energy ion scattering, which showed no Zr signal after annealing, and XPS, which proved the exclusively bimetallic state of Zr^0 at a binding energy of 179.6 eV [2,3].

For the subsequent measurements under DRM conditions, three different experimental setups, two of them being synchrotron endstations, were used:

<u>Setup 1:</u> *Surface preparation and -analysis chamber with attached high-pressure batch reaction cell*

The UHV system with attached all-quartz recirculating batch reactor, described in detail in [4], is designed for quantitative catalytic/kinetic studies up to 1 bar on polycrystalline foils, detecting products by online MS analysis (HP GC-MS System G1800A) via a capillary leak and/or by conventional GC-MS analysis via column injection. MS signals of CH_4, CO_2 and CO (m/z= 15+16, 44 and 28) were externally calibrated and corrected for fragmentation. Hydrogen can be quantified using an additional detection line to a differentially pumped Balzers QMA 125 quadrupole mass spec. Ex-situ surface analysis was performed using a XPS/AES/LEIS spectrometer (Thermo Electron Alpha 110) and a twin Mg/Al anode X-ray gun (XR 50, SPECS). All DRM reactions were conducted with initial partial pressures of CH_4 : CO_2 = 50 mbar : 50 mbar. The reaction cell was backfilled with pure He to a total pressure of 970 mbar, in order to achieve efficient gas intermixing via recirculation and fast heat transfer to the sample via thermal conductivity. For DRM catalysis, the reactor was heated at a constant linear rate of 25 K/min to the final temperature of 800 °C and then kept isothermally at this temperature for ~30 min. From the product partial pressures vs. time plots, the reaction rates were obtained by differentiation and are given in partial pressure change per minute [mbar/min].

<u>Setup 2:</u> *Ambient pressure (AP)-XPS spectrometer for in-situ catalyst characterization*

The vacuum chamber operated at HZB/BESSY II (at beamline ISISS-PGM) allowed us to perform *in-situ* photoelectron spectroscopy up to 1 mbar total reactant pressures [5]. The subsurface Zr^0/Pd-foil and bulk Pd_xZr_y precatalyst samples were positioned inside the high-pressure analysis chamber ~2 mm away from a 1 mm aperture, which is the entrance to the differentially-pumped electrostatic lens system separating gas molecules from photoelectrons focused toward the SPECS hemispherical analyzer. Binding energies were referred to the Fermi edge recorded after each scan. The temperature was measured by a K-type Ni/NiCr thermocouple directly spot-welded to the sample and temperature-programmed heating was done by an IR laser from the rear. The subsurface alloy sample was prepared in Setup 1 as described above, and then transferred to BESSY II under inert dry gas phase conditions provided inside the transportable sample load-lock setup. For final transfer to the AP-XPS chamber, it was briefly exposed to ambient pressure conditions at ~293 K. Nevertheless, no signs of oxidative Zr segregation were visible thereafter, even at low photoelectron kinetic energies (i.e. at high surface sensitivity). The bulk intermetallic samples were also transferred to BESSY II in inert gas after the above-described melt preparation, but due to the coexistence of Zr^0 and Pd^0 metal in the surface, already the initial (pre-DRM) sample showed a considerable degree of oxidative segregation toward ZrO_2 and Pd^0 after the contact to the ambient (cf. Fig. S1).

Monochromator control allows to choose photon energies corresponding to common kinetic energies of the ejected photoelectrons (typically 400 eV in this work) for all monitored core-level photoemission peaks in order to extract information from a constant information depth and to yield the same attenuation of the photoelectrons through the gas phase. Photoelectrons are collected in the direction normal to the surface at constant pass energy of 10 eV. Photoemission peak intensities are corrected for the respective photon flux at a given photon energy. Since the BESSY II synchrotron operates in top-up mode (constant ring current), no additional correction for the ring current was required. Since all photoemission peaks were collected at the same kinetic energy of photoelectrons, the overall attenuation effects were the same for all-core levels and, thus, cancel out in quantification routines.

Setup 3: The bulk structural changes of the melt-prepared intermetallic PdZr sample were investigated in-situ during heating under DRM conditions by synchrotron XRD [6] at Beamline 12.2.2 (Advanced Light Source ALS, Lawrence Berkeley National Labs LBNL, California). X-ray diffraction was performed with a monochromatic beam (30µm spot size, 0.5172 A, MAR345 detector, 300sec total acquisition time) in transmission mode with a beam energy of 24 keV, just below the Pd K edge at 24.3503 keV, to achieve sufficient pattern intensity. The Fit2d software [7] was used for calibration and integration. Detector-sample distance and wavelength were determined using NIST standard reference material 660b LaB_6. The sample was powder-ground before being transferred to a 700 µm quartz capillary. The desired gas atmosphere was achieved by injection of a 1:1 CH_4:CO_2 mixture (flow rate: 1 ml/min each, ambient pressure), and a heating rate of 20 K/min to 800 °C was applied for DRM testing.

Details of XPS analysis:

The XPS data of Setups 1 and 2 were analysed using the CasaXPS software program, version 2.3.16 Pre-rel 1.4 (Casa Software Ltd) [8]. For quantification and peak fitting a Shirley background was applied to all spectra and the associated Scofield relative sensitivity factors were considered for quantification. Peak fitting of the Pd3$d_{5/2}$ and C1s peaks was done using a weighted sum of Gaussian and Lorenzian peak shapes with a GL(30) contribution. Asymmetric parameters were used for Pd3$d_{5/2}$ fitting of Pd^0 (GL(30)T(1.3)) and Pd_xC (GL(30)T(1.5)) as well as for C1s fitting of graphite (GL(30)T(1.1)) and Pd_xC (GL(30)T(1.3)). Regarding the BE component assignment, all Pd3$d_{5/2}$ spectra show a component at a BE of 333.5 eV (labelled as O1s 2nd harm.) which is caused by additional frequency-doubled excitation of the O1s signal due to the second diffraction order of the synchrotron radiation at the plane grating monochromator. Deconvolution of all Pd3$d_{5/2}$ spectra involved the metallic Pd component at a BE of 335.0 eV, and the carbidic Pd_xC component at a BE of 335.6 eV [9,10].

Different C1s components are considered in all C1s spectra, namely: carbonates and carbon-oxygenates above 287 eV [11], and C_xH_y species at BE's of ~285.4 eV [11–13]. The component at a BE of 283.0 eV is assigned to interstitial/ carbidic C in Pd_xC [14].

Electron attenuation lengths were taken from the NIST database SR 82 [15]. The orbital asymmetric parameters were taken from the ELETTRA online database [16].

Supplemental Results

S1: Schematic representation of the two types of employed model catalysts and their distinct chemical and structural development under dry reforming conditions.

The simplified schematic representation of the “inverse” pre-catalyst is based on a fcc (100) lattice plane containig Zr^0 atoms in the subsurface region. The subsurface intermetallic state has been verified in setup 1 by a combination of XPS and LEIS, as well as the “ZrO_2 islands on top” state after oxidative segregation under reaction conditions.

The schematic lattice of the bulk intermetallic pre-catalyst does not reflect the polycrystalline Pd_2Zr + Pd_3Zr phase mixture used in reality. Under DRM conditions, crystalline Pd^0 is formed, as well as t-ZrO_2, which is also not represented with its actual 3D crystal structure. Both newly formed phases - Pd and t-ZrO_2 – form a nanocrystalline conglomerate on top of the polycrystalline Pd_xZr_y substrate. The particle size and the lattice constant of Pd^0 were estimated to ~7.5 nm/ 3.914 Å in-situ during DRM at 800°C and to 10.3 nm/ 3.898 Å after DRM and subsequent O_2 oxidation.

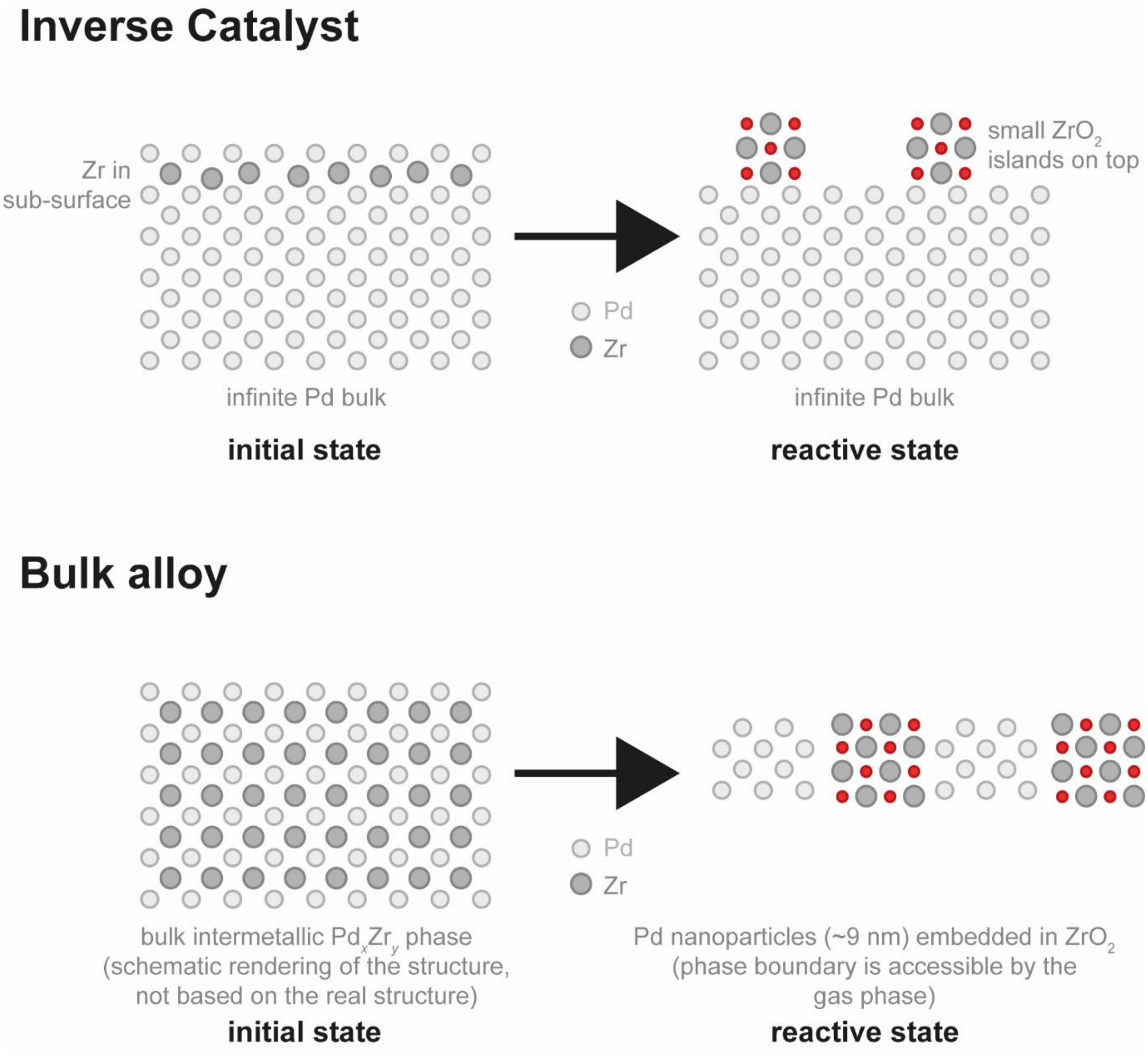

Fig. S1: Simplified schematic representation of the Zr^0 subsurface alloy vs. bulk intermetallic pre-catalysts and their transformations under realistic dry reforming conditions.

S2: Pre-DRM state of the bulk-intermetallic PdZr precursor. Despite the dominance of Zr^{+4} species in the Zr3d region, the intermetallic Zr^0 component is clearly visible at a BE of 179.6 eV [2,3]. Already the short contact to the ambient during transfer to the AP-XPS chamber caused oxidative segregation of the bimetallic toward a top layer of ZrO_2 and metallic Pd. The $Pd3d_{5/2}$ region is superimposed by the second harmonic contribution of the O1s signal at a nominal BE of 333.5 eV, otherwise only metallic Pd is observed at a BE of 335.0 eV. In the C1s region a certain amount of graphite-type C is already present before DRM, with minor contributions of C_xH_y species and C-oxygenates [11,13,17].

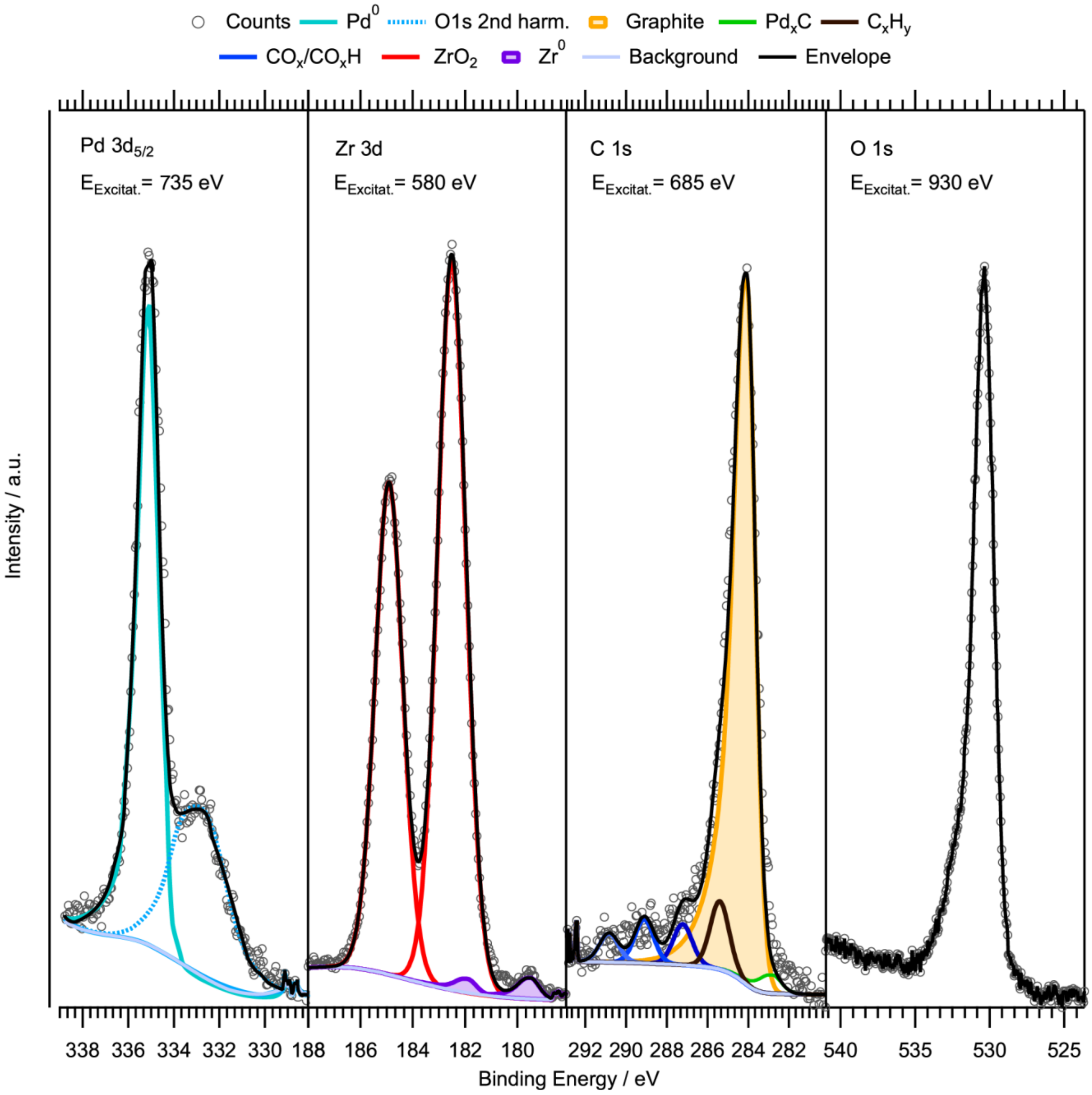

Fig. S2: $Pd3d_{5/2}$, Zr3d, C1s and O1s core level regions of the "precatalytic" bulk-intermetallic PdZr surface. The kinetic energy of the photoelectrons was adjusted to 400 eV for all regions via photon energy variation.

S3: Changes of Pd lattice parameter and particle size with time observed by in-situ XRD during reoxidation of the post-DRM sample in a flow of 2 ml/min clean oxygen at ambient pressure and 600°C. Between 25 and 35 min exposure to O_2, a lowering of the lattice constant by ~0.016 Å takes place. We assume that the partial oxidation of Pd metal toward PdO is accompanied by simultaneous carbon depletion of the Pd bulk, thus leading to a smaller lattice parameter.

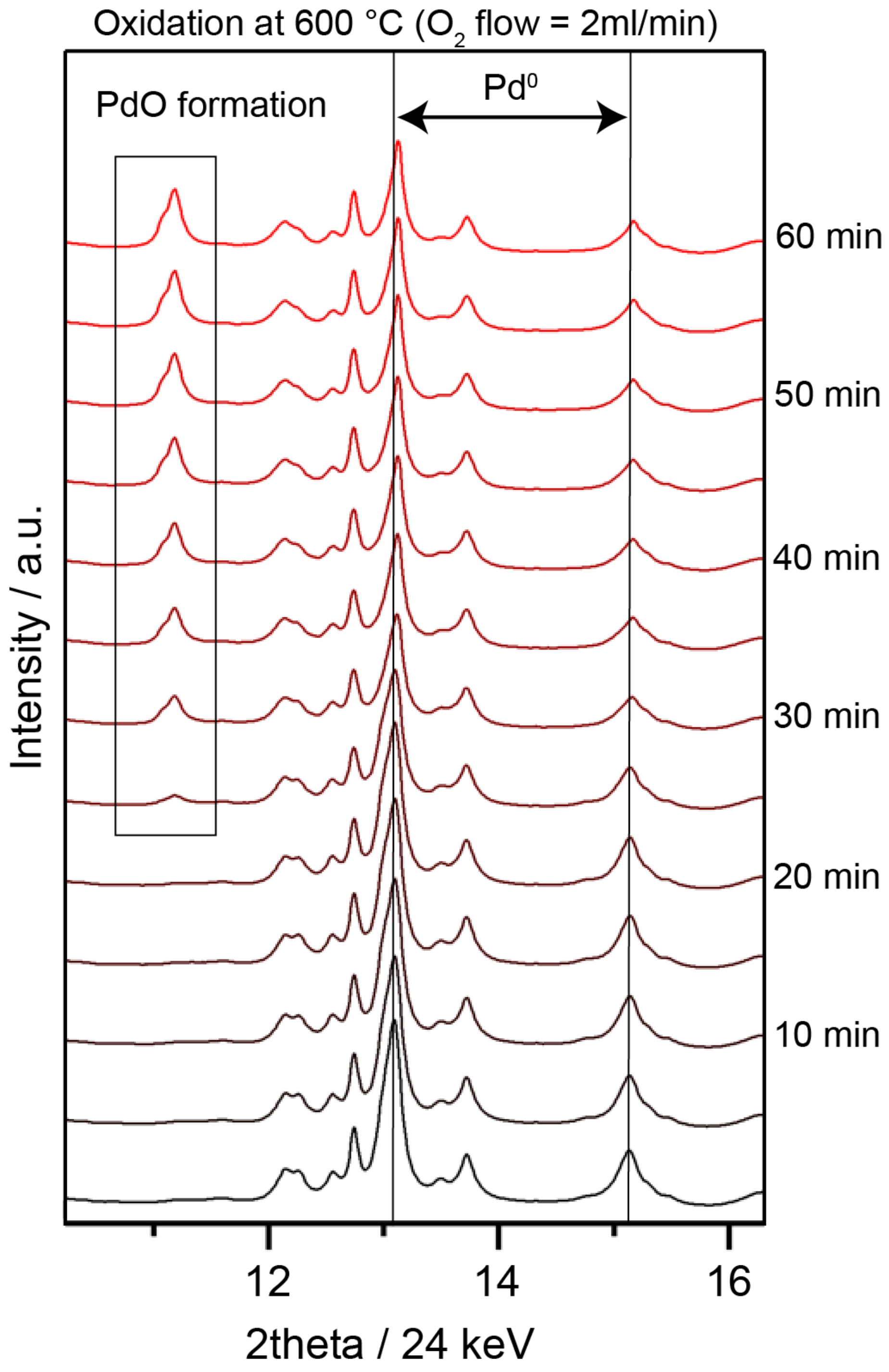

Fig. S3: Time-resolved in-situ XRD spectra of the Pd/PdO reflex region of the initially bulk-intermetallic PdZr catalyst during reoxidation in 2 ml/min oxygen at 600°C after DRM at 800 °C, taken in order to check the reversibility of the DRM-induced structural changes and of the carbon deposition.

S4: Temperature programmed DRM reaction rate profiles obtained on the subsurface Zr^0-Pd foil precatalyst versus single phase ZrO_2 film and clean Pd foil. The reaction conditions were identical to those of Figure 2 in the main paper. Only a slight promotion of DRM activity was observed on the subsurface Zr^0-Pd foil precatalyst relative to phase-pure ZrO_2. Pure Pd is hardly active, anyway.

The CVD preparation of sub-monolayer ZrO_xH_y islands, and in due course, of our “inverse” subsurface-Zr^0-intermetallic pre-catalysts on bulk Pd foil, was originally motivated by a potentially scalable promotional role of variable phase boundary dimensions on the DRM activity. What actually came out was initially surprising, namely that all these “inverse” systems were only slightly more active than the sum of their individual oxidic and metallic surface fractions. The explanation for strong DRM promotion given in the main paper for the bulk-intermetallic precursor requires sufficiently high concentrations of reactive dissolved C arriving through the metallic phase toward the PB, which is obviously not the case on the infinite bulk „inverse“ models on Pd foil. Thus, also changes of the initial bimetallic Zr^0 amount between 5% and 90% led to only minor differences in the observed activities. In practice, these tiny differences were not scalable with the in-situ DRM induced PB dimensions for several reasons:

-poor signal to noise ratio due to low overall activity, together with too small activity differences. Given our experimental error bars at these rather low total rates, we cannot quantify the relative contributions of the two surface regions relative to the PB sites.

-non-scalable amounts of reactive dissolved C in the Pd foil bulk (which are both time-, temperature and oxide-coverage-dependent)

-non-scalable island density of re-segregated ZrO_xH_y from the bimetallic precursor state, thus the exact amount of phase-boundary sites cannot be quantified. The exact nanoscopic size-distribution of the in-situ segregated islands is not known, and thus, a normalization of these tiny rate differences to well-understood and –quantified phase-boundary dimensions is actually impossible in our view. One would have to employ in-situ microscopic techniques at the atomic scale under realistic DRM conditions, which is experimentally out of reach.

-even if the latter experiment was possible, the very small activity differences would not allow for a clear scaling relation between PB sites and activity differences, for the above mentioned reasons.

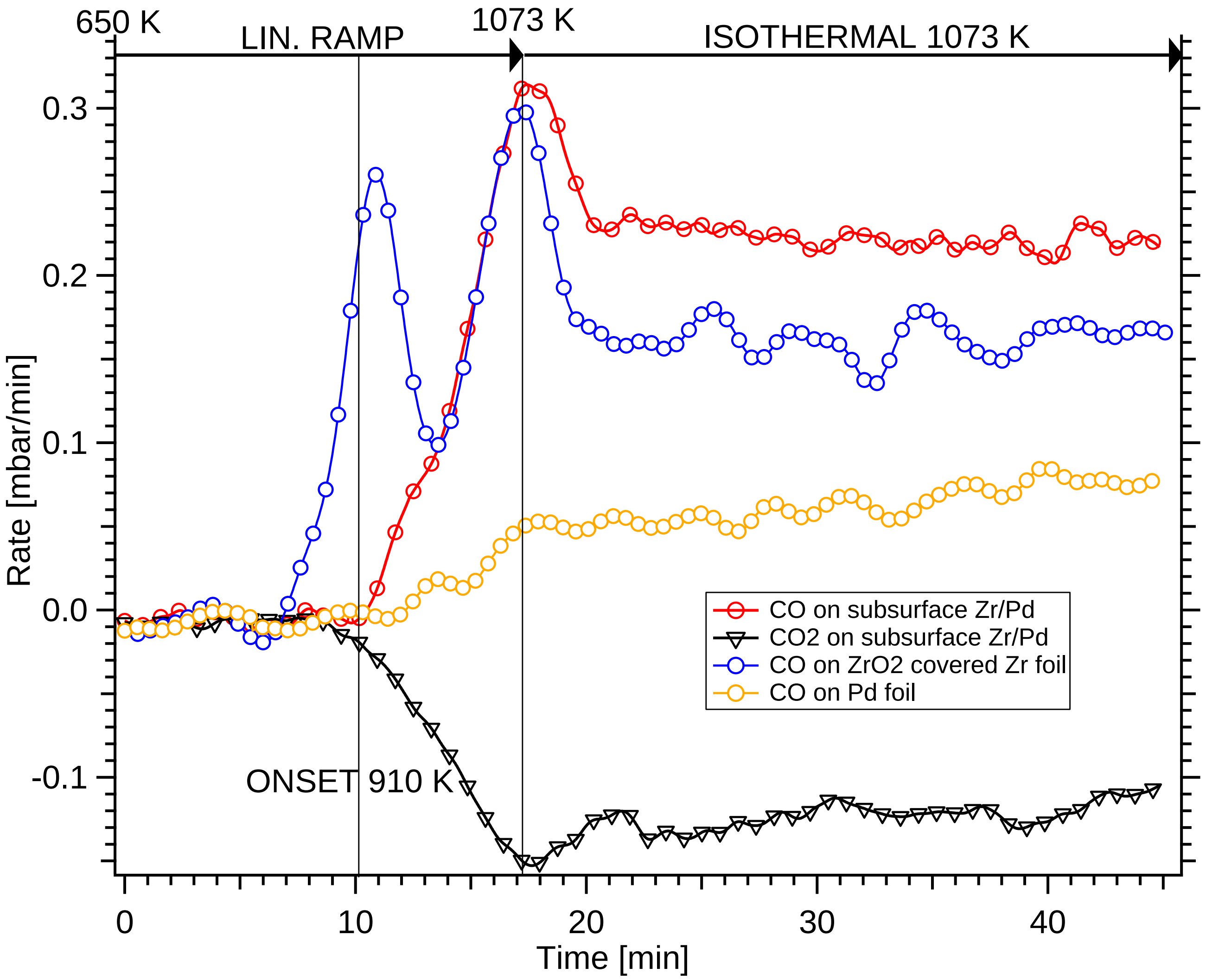

Fig. S4: Temperature programmed DRM reaction rate profile obtained on the subsurface Zr^0-Pd foil precatalyst in 50 mbar CH_4 + 50 mbar CO_2 vs. clean Pd and ZrO_2. Heating rate: 25 K/sec, isothermal reaction for 30 min at 800 °C.

S5: Relative intensity variations of the C_{bulk}, $C_{graphite}$ and $C_{oxygenate}$ C1s intensities with XPS probe depth. We note that also the oxygenate region exhibits an apparent intensity increase at higher photon energies. It is safe to say that a volume-dissolved species such as C_{bulk} should exhibit reduced signal attenuation with increasing hν/probe depth/photoelectron kinetic energy, in particular relative to surface-located graphene/graphite layers. In analogy, the oxygenate C1s intensity appears to be less attenuated than $C_{graphite}$. According to scheme S1, the corrosion of the bulk intermetallic leads to a „conglomerate" of nano-sized Pd^0 and t-ZrO_2 domains, which inherently leads to a large amont of „buried" phase boundary sites. An enhanced concentration of oxygenate species at these sites would result in a „quasi-3D" depth distribution perpendicular to the outer (geometrical) surface, and thus to a similar response to photon energy variation as for C_{bulk}. We emphasize that this should be definitly seen as a speculation, as the poor data quality and the ambiguity of the background subtraction in the oxygenate C1s region does not allow for reliable assignments of intensity trends.

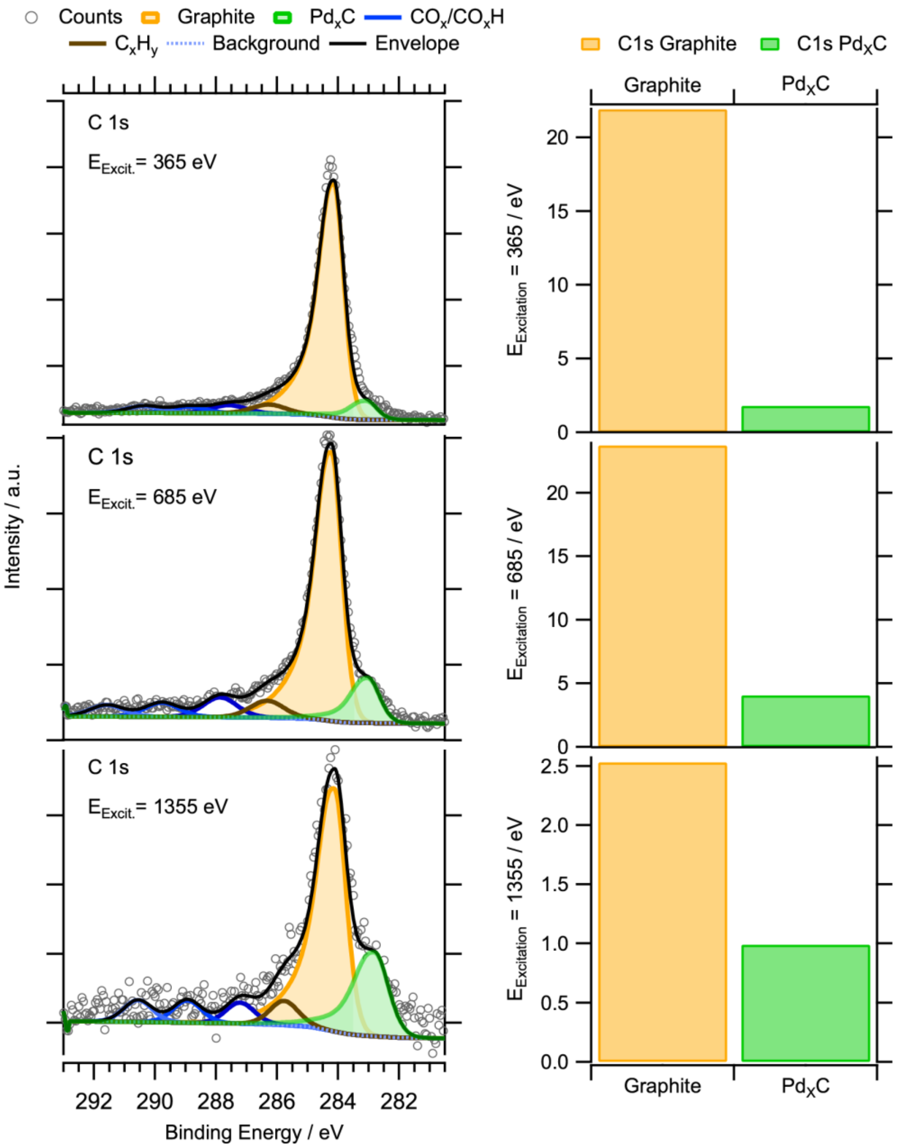

Fig. S5: In-situ depth profiling of the C1s signal under isothermal DRM conditions at constant reactant pressures. T = 700°C, $p(CH_4)$: $p(CO_2)$ = 0.15 mbar : 0.15 mbar.

S6: Comparison of the initial/pre-DRM and in-situ clean-off carbon concentrations in pure CO_2 on the initially bulk-intermetallic catalyst. After exposure to clean CH_4, which led to the carbon level depicted in the left spectrum of Figure 3 A), the exposure to 0.3 mbar clean CO_2 was kept for 150 min at a temperature of ~740 °C. We note that the C clean-off became very slow - slower than for the CH_4- (or also DRM-) induced $C_{graphite}$ fraction - at a concentration level slightly below the precatalyst C-level, indicating that the remaining "ambient-induced" C-fraction is kinetically less accessible. After 150 min, the experiment had to be cancelled because of limited beamtime. From the peak areas of Fig. S6, values for 11.3 atom % C on the initial and 8.5 atom % C on the in-situ-CO_2 states were derived via a standard quantification routine assuming homogeneously mixed elements. We can hardly speculate on the chemical nature, location and diffusional mobility of these ambient exposure-induced C-species, and only roughly estimate their remaining life time in clean CO_2. Based on an intensity decrease of ~2-3% within roughly 2-3 hours, it would take at least 6 hours to remove the largest fraction. A possible explanation for the low reactivity could involve their location in more remote zones with respect to the active PB region and/or otherwise impaired diffusional properties.

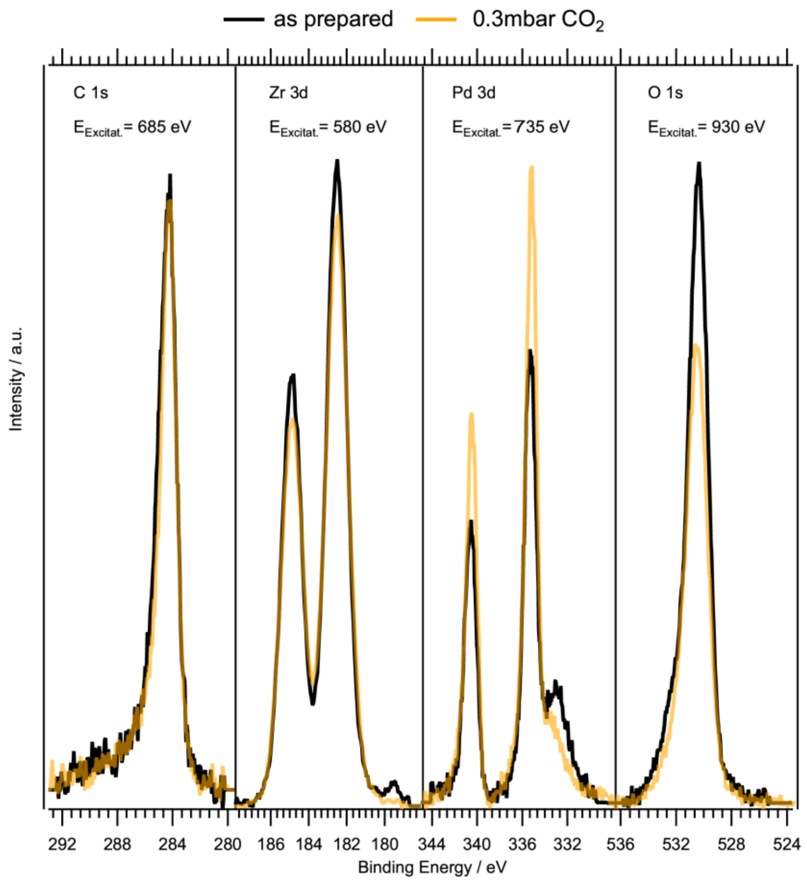

Fig. S6: Relative intensities of the C1s, Zr3d, Pd3d and O1s regions, determined at a common photoelectron kinetic energy of 400 eV. Black traces: precatalyst before DRM; dark yellow-coloured traces: during CO_2 clean-off in 0.3 mbar after 150 min at 740°C. Both sets of spectra were recorded at the same total pressure in order to establish comparable gas-phase absorption conditions for the photoelectrons.

S7: In-situ AP-XPS spectra on the initial subsurface Zr^0-Pd foil catalyst under DRM conditions. Intensity trends associated with changing gas phase conditions are depicted in the bar viewgraph at the right side. Complete carbon clean-off is observed in pure CO_2; the carbon present after growth in clean CH_4 appears to be rather located on top of the ZrO_2 islands, which are formed in-situ under DRM conditions. A lower and time-independent steady-state carbon concentration than that growing in pure CH_4 is established in the 1:1 DRM reactant mixture.

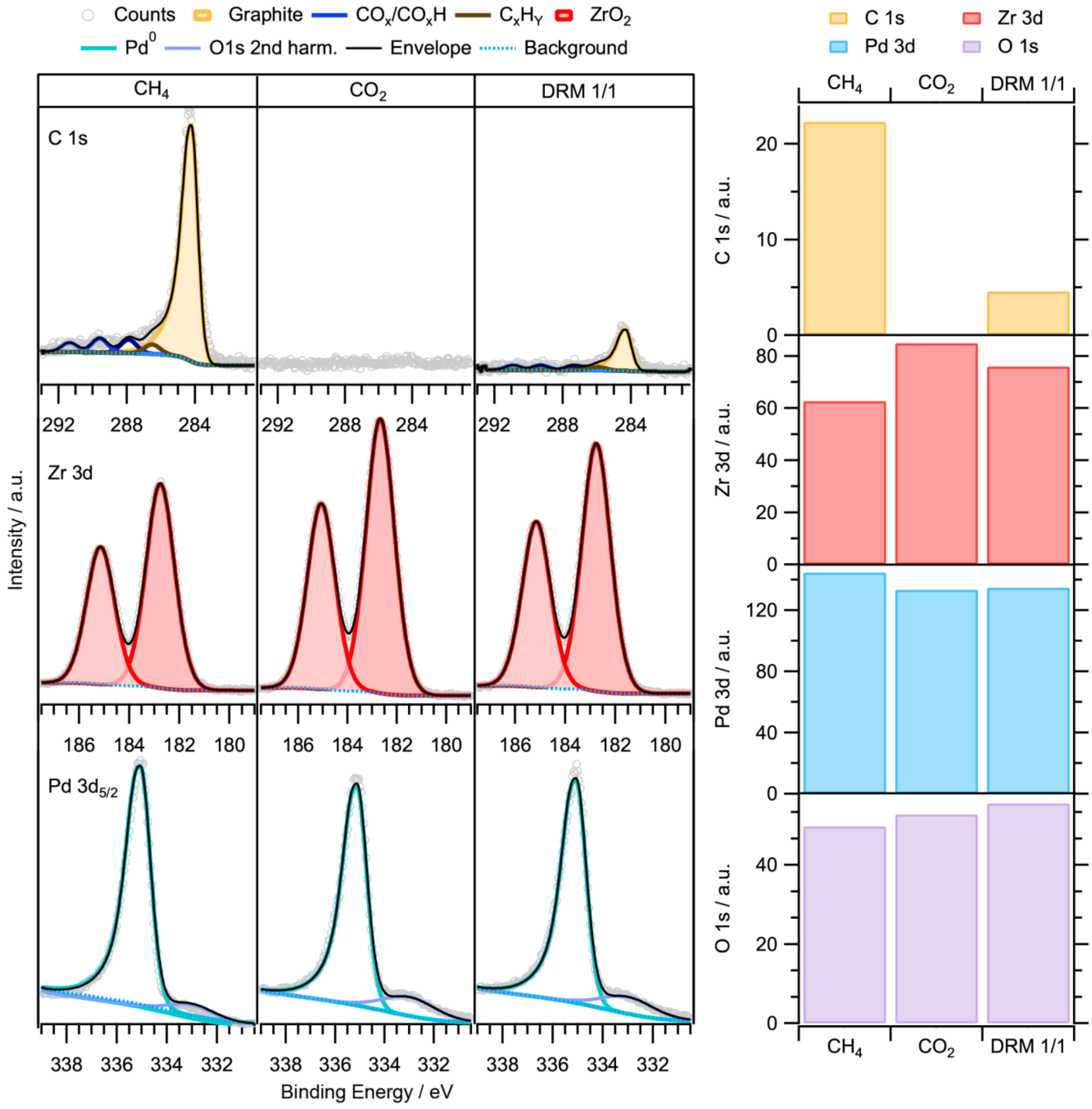

Fig. S7: C1s, Zr3d and Pd3d$_{5/2}$ AP-XPS spectra recorded in situ at a common kinetic energy of 400 eV at ~700°C on the initial subsurface Zr^0-Pd foil catalyst. Left panel/left side: 0.3 mbar pure CH_4; middle panel/left side: 0.3 mbar pure CO_2; right panel/left side: 0.15 mbar CH_4 + 0.15 mbar CO_2. Right side: bar viewgraph of intensity trends.

S8: Change of steady-state C_{bulk} and $C_{graphite}$ components as a function of temperature under in-situ DRM conditions. Between 600 and ~700°C, both components show increasing intensities, beyond ~700°C they both decrease, whereby the relative response of C_{bulk} appears to be generally stronger. Up to ~700°C this may be caused by the increase of C solubility and the simultaneously accelerated CH_4 decomposition rate with increasing temperature, meaning that the Pd particles exhibit enhanced (net) carbon dissolution. Beyond ~700°C, thermodynamics generally favour the C depletion, since ΔG of $CH_4 \rightarrow 2H_2 + C_s$ decreases with increasing T, but at a lower rate than ΔG of the inverse Boudouard reaction $CO_2 + C_s$ -> 2CO. The temperature in which the rate of C_s generation exceeds that of C oxidation by CO_2 is around 725 °C, as already reported in [18]. A complementary kinetic model accounting for increasingly fast C_{bulk} depletion toward CO via the PB at higher temperatures is proposed to explain this trend. A detailed numerical balancing of PB clean-off rates of C_{bulk}, C_{bulk} supply both via graphite redissolution and continuous carbon supply via CH_4 from the gas phase, has not yet been attempted.

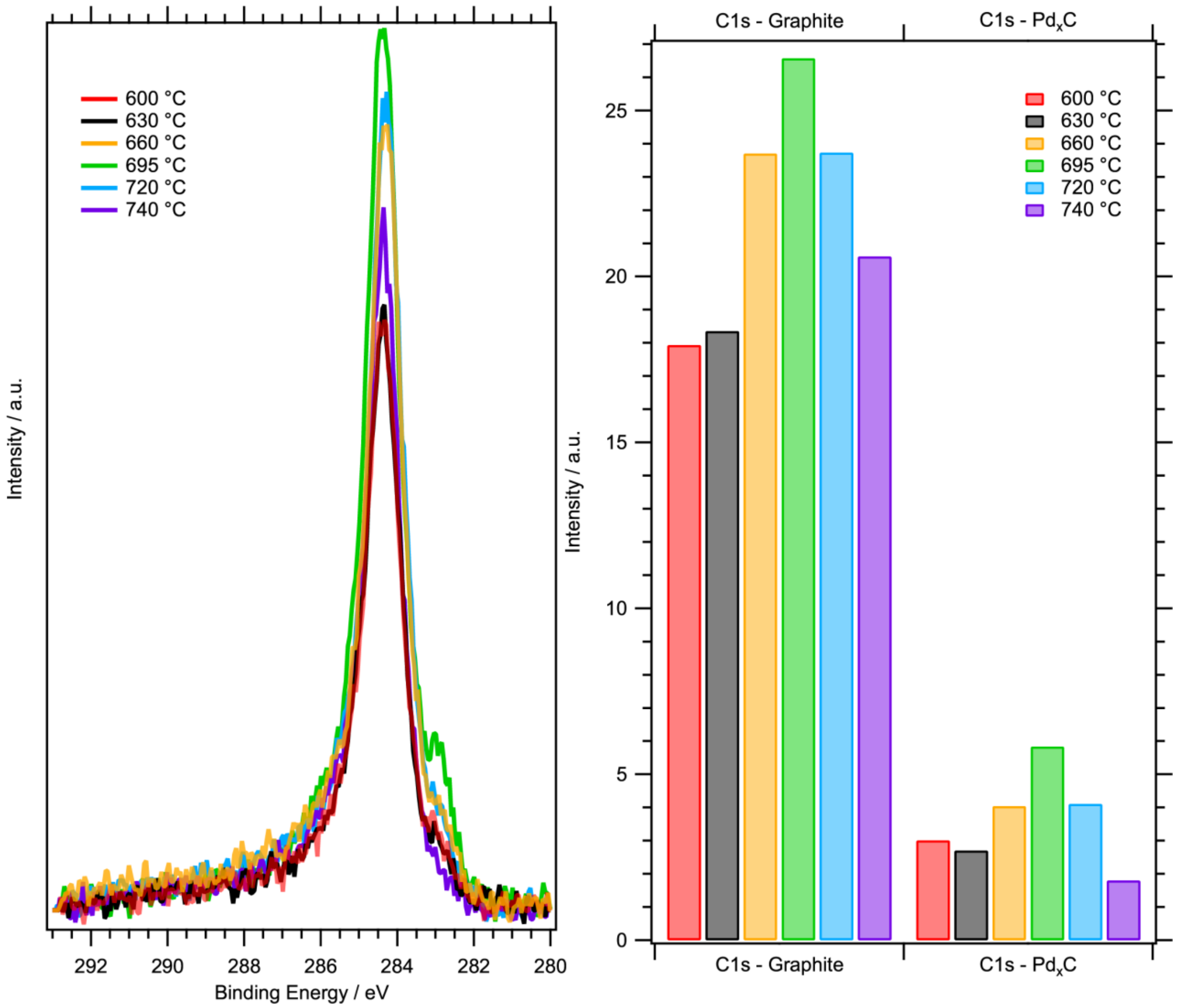

Fig. S8: Response of the steady-state C_{bulk} and $C_{graphite}$ intensities to temperature changes between 600 and 740°C. Constant DRM pressure conditions: 0.15 mbar CH_4 + 0.15 mbar CO_2. Photoelectron kinetic energy 400 eV, hν = 685 eV.

Supporting Literature:

[1] L. Mayr, X.-R. Shi, N. Köpfle, C. A. Milligan, D. Y. Zemlyanov, A. Knop-Gericke, M. Hävecker, B. Klötzer, S. Penner, *Phys. Chem. Chem. Phys.* **2016**, *18*, 31586.

[2] N. Köpfle, L. Mayr, P. Lackner, M. Schmid, D. Schmidmair, T. Götsch, S. Penner, B. Kloetzer, *ECS Trans.* **2017**, *78*, 2419.

[3] H. Li, J.-I. J. Choi, W. Mayr-Schmölzer, C. Weilach, C. Rameshan, F. Mittendorfer, J. Redinger, M. Schmid, G. Rupprechter, *J. Phys. Chem. C* **2015**, *119*, 2462.

[4] L. Mayr, R. Rameshan, B. Klötzer, S. Penner, C. Rameshan, *Rev. Sci. Instrum.* **2014**, *85*, 55104.

[5] H. Bluhm, M. Hävecker, A. Knop-Gericke, M. Kiskinova, R. Schlögl, M. Salmeron, *MRS Bull.* **2007**, *32*, 1022.

[6] a) L. Schlicker, A. Doran, P. Schneppmüller, A. Gili, M. Czasny, S. Penner, A. Gurlo, *Rev. Sci. Instrum.* **2018**, *89*, 33904; b) A. Doran, L. Schlicker, C. M. Beavers, S. Bhat, M. F. Bekheet, A. Gurlo, *Rev. Sci. Instrum.* **2017**, *88*, 13903.

[7] A. P. Hammersley, S. O. Svensson, M. Hanfland, A. N. Fitch, D. Hausermann, *High Pressure Res.* **1996**, *14*, 235.

[8] *CasaXPS Version 2.3.16 Pre-rel 1.4*, Casa Software Ltd, **2011**.

[9] O. Balmes, A. Resta, D. Wermeille, R. Felici, M. E. Messing, K. Deppert, Z. Liu, M. E. Grass, H. Bluhm, R. van Rijn et al., *Phys. Chem. Chem. Phys.* **2012**, *14*, 4796.

[10] D. Teschner, J. Borsodi, A. Wootsch, Z. Révay, M. Hävecker, A. Knop-Gericke, S. D. Jackson, R. Schlögl, *Science* **2008**, *320*, 86.

[11] A. Wolfbeisser, B. Klötzer, L. Mayr, R. Rameshan, D. Zemlyanov, J. Bernardi, K. Föttinger, G. Rupprechter, *Catal. Sci. Technol.* **2015**, *5*, 967.

[12] N.M. Rodriguez, P.E. Anderson, A. Wootsch, U. Wild, R. Schlögl, Z. Paál, *J. Catal.* **2001**, *197*, 365.

[13] D. N. Belton, S. J. Schmieg, *J. Vac. Sci. Technol., A* **1990**, *8*, 2353.

[14] B. S. Ahn, S. G. Jeon, H. Lee, K. Y. Park, Y. G. Shul, *Appl. Catal., A* **2000**, *193*, 87.

[15] C. J. Powell and A. Jablonski, *NIST Electron Effective-Attenuation-Length Database SRD 82,*, Institiue of Standards and Technology, Gaithersburg, **2011**.

[16] J. J. Yeh, *Atomic Calculation of Photoionization Cross-Sections and Asymmetry Parameters*, Gordon and Breach Science Publishers, Langhorne, PE, USA, **1993**.

[17] a) Z. Bastl, *Collect. Czech. Chem. Commun.* **1995**, *60*, 383; b) G.J. Kovács, I. Bertóti, G. Radnóczi, *Thin Solid Films* **2008**, *516*, 7942; c) N. Laidani, L. Calliari, G. Speranza, V. Micheli, E. Galvanetto, *Surf. Coat. Technol.* **1998**, *100-101*, 116.

[18] M. C. J. Bradford, M. A. Vannice, *Catal. Rev. Sci. Eng.* **1999**, *41*, 1.

Zusatzinformation

Methodensektion

Details der Probenpräparation

Die Synthese der volumens-intermetallischen PdZr-Probe wurde mittels Widerstandsheizung kleiner aufeinandergestapelter Stücke von jeweils reinem Pd- und Zr-Blech unter Vakuumbedingungen ($1x10^{-7}$ mbar) in einem Ta-Schiffchen durchgeführt. Das nominelle Verhältnis Pd:Zr betrug dabei 2:1. Oberhalb des Schmelzpunktes von reinem Pd (1828 K) wurde eine spontane exotherme Reaktion zwischen Pd und Zr beobachtet, die zu einer intermetallischen Schmelze führte. Die Heizung wurde daraufhin abgeschaltet und die Probe durch Abkühlung rekristallisiert. Die entstandene Mischung aus volumens-intermetallischen Pd-Zr Phasen wurde entsprechend charakterisiert, wie in Abbildung 1 des Haupttexts gezeigt.

Als Referenzkatalysator für die Dünnfilm-Intermetallprobe wurde eine ultrareine Pd Folie (Goodfellow, Reinheit 99.95%, Dicke: 0.1 mm, Größe etwa 7 cm^2) verwendet. Präparation und Charakterisierung der Oberfläche beinhaltete zyklisches Anwenden von Ar Sputtern und thermisches Heizen ($6.0x10^{-5}$ mbar Ar, 2 keV, 1 µA Probenstrom; T = 700 °C), bis XPS und Auger Spektren keine Verunreinigungen mehr zeigten.

Für die Präparation des oberflächennahen Pd^0Zr^0-Intermetallzustands wurde als Präkursor Zr-tert.-Butoxid (ZTB, Sigma Aldrich, Reinheit: 99.999%) verwendet, das während der chemischen Gasphasenabscheidung über ein Ventil direkt auf die Pd-Folie dosiert wurde. Hierfür wurde die UHV Anlage, im folgenden „Setup 1“ genannt, verwendet. Nach Exposition bei einem

Druck von $5.0x10^{-6}$ mbar für 4 min., entsprechend $1.2x10^{-3}$ mbar*sec, wurde eine Oberflächenbedeckung von ~ 0.5 ML Zr via XPS berechnet. Details der Quantifizierung sind in Ref. [1] nachzulesen. Der resultierende hydroxylierte $Zr^{4+}O_xH_y$ Zustand auf dem Pd^0 Substrat wurde dann unter ausgezeichneten UHV Bedingungen thermisch auf ~430 °C geheizt. Nur bei Drücken unterhalb $5x10^{-10}$ mbar wurde eine 100%ige Umwandlung zu einem oberflächennahen Zr^0-Zustand beobachtet. Dies repräsentiert eine deutliche Verbesserung zur Synthese des teilweise oxidierten Präkatalysators von Ref. [1]. Der 100%ige oberflächennahe Intermetallzustand wurde durch eine Kombination aus Niederenergie-Ionenstreuung (kein Zr Signal nach thermischem Heizen) und XPS (es wurde nur der bimetallischer Zr^0 Zustand bei einer Bindungsenergie von 179.6 eV beobachtet) verifiziert [2,3].

Für die Messungen unter Trockenreformierbedingungen wurden drei Setups, zwei von diesen synchrotron-basiert, verwendet.

Setup 1: Oberflächenpräparation und –Analyse mit Hochdruck-Katalysereaktor

Das verwendete UHV System mit einem Hochdruck-Katalysereaktor aus Quarzglas, beschrieben im Detail in Ref. [4], ist speziell auf quantitative katalytische bzw. kinetische Studien bei Drücken bis 1 bar auf polykristallinen Folien ausgerichtet, und ermöglicht Online-Produkt-Detektion per Gaschromatographie/Massenspektrometrie-Kopplung (HP GC-MS G1800A) per Kapillarventil und/oder per konventioneller GC/MS Analyse mittels Säuleninjektion. Die Signale von Methan, Kohlendioxid und Kohlenmonoxid (m/z = 15+16, 44 und 28) wurden extern kalibriert und bezüglich Fragmentierung korrigiert. Wasserstoff kann mittels zusätzlicher Online-Detektion mit einem differentiell gepumpten Quadrupol-MS (Balzers QMA 125) quantifiziert werden. Ex-situ Oberflächencharakterisierung wurde mittels eines XPS/Auger/Niederenergie-Ionenstreuungs-Spektrometers (Thermo Electron Alpha 110) und einer Mg/Al Röntgenanode (XR 50, SPECS) durchgeführt. Alle Trockenreformier-Experimente wurden mit anfänglichen Partialdrücken von Methan : Kohlendioxid = 50 mbar: 50 mbar durchgeführt. Der Reaktor wurde jeweils mit reinem He auf 970 mbar Totaldruck aufgefüllt, um einerseits eine effiziente Gas-Durchmischung, andererseits einen verbesserten Wärmetransport zur Probe zu gewährleisten. Lineare Heizraten von 25 K/min auf die finale Temperatur von 800 °C, gefolgt von einer isothermen Periode bei dieser Temperatur für 30 min, wurden verwendet. Aus den Partialdruck- vs. Zeit- Diagrammen wurden die Reaktionsraten durch Differentiation bestimmt. Diese sind im Folgenden als Änderung der Partialdrücke per Minute [mbar/min] angegeben.

Setup 2: Röntgenphotoelektronenspektrometer bei Umgebungsbedingungen für die *in-situ* Charakterisierung

Die Vakuumkammer bei HZB/BESSY II (Endstation ISISS-PGM) erlaubt Photoelektronenspektroskopie bis 1 mbar Druck [5]. Die Proben wurden in der Hochdruck-Analysekammer ~ 2 mm von einer 1 mm Apertur positioniert, die als Eintrittsblende in das differentiell gepumpte elektrostatische Linsensystem fungiert, welches die Gasmoleküle von den zum SPECS Halbkugel-

Analysator fokussierten Photoelektronen trennt. Bindungsenergien wurden auf die mit jedem Scan aufgezeichnete Fermi-Kante referenziert. Die Temperaturmessung erfolgte durch ein K-Typ Ni/NiCr Thermoelement, das direkt an die Probe angepunktet wurde. Die Heizung wurde durch einen IR-Laser von der Probenrückseite gewährleistet. Der oberflächennahe Intermetallzustand wurde wie bei Setup 1 diskutiert, hergestellt und zu BESSY II unter inerten Bedingungen mittels eines transportablen Load-Locks transferiert. Der finale Transfer zur Kammer involvierte einen kurzen Kontakt mit der Umgebungsluft bei etwa 293 K. Nichtsdestoweniger wurde keine oxidative Zr Segregation beobachtet, vor allem bei niedrigen Photoelektronenenergien, d.h. hoher Oberflächensensitivität. Der Transfer der volumens-intermetallischen PdZr-Probe erfolgte nach entsprechender Synthese analog, allerdings zeigte sich durch die gleichzeitige Anwesenheit von Zr^0 und Pd^0 in der Oberfläche ein entsprechender Anteil oxidativer Segregation zu ZrO_2 und Pd^0 bereits nach kurzem Kontakt mit Umgebungsbedingungen (cf. Abbildung S1).

Über die Monochromatoreinstellung konnten die Photonenenergien und in der Folge die kinetischen Energien der Photoelektronen (typischerweise 400 eV) für alle aufgezeichneten XPS-Regionen ausgewählt werden. Dies ermöglicht eine Analyse mit einheitlicher Informationstiefe, und eine identische Abschwächung der Photoelektronen durch die Gasphase. Die XPS-Intensität wurde normal zur Oberfläche bei einer konstanten Passenergie von 10 eV gemessen. Peakintensitäten wurden auf den bereits kalibrierten Photonenfluss bei einer gegebenen Photonenenergie normiert. Da BESSY II mit konstantem Ringstrom-Modus betrieben wird, ist eine zusätzliche Normalisierung der Intensitäten auf den Ringstrom nicht notwendig. Da alle Photoemissionspeaks bei der gleichen kinetischen Energie der Photoelektronen gesammelt wurden, fallen energiespezifische Abschwächungseffekte (die dadurch für alle XPS-Niveaus identisch sind) bei der Quantifizierung heraus.

Setup 3: Die Strukturänderungen der volumens-intermetallischen PdZr-Probe wurden unter Trockenreformierbedingungen per in-situ Synchrotron-Röntgenbeugung [6] am Strahlrohr 12.2.2 (Advanced Light Source, ALS, Lawrence Berkeley National Labs LBNL, Kalifornien, USA) durchgeführt. Hierzu wurde ein monochromatischer Strahl (30 µm Strahlgröße, 0.5172 A, MAR345Detektor, 300 sec totale Aufzeichnungszeit) in Transmissionsmodus mit einer Strahlenergie von 24 keV - direkt unterhalb der Pd K Kante bei 24.3503 keV zur Erhöhung der Intensität der Diffraktogramme - verwendet. Kalibration und Integration der Daten erfolgte durch das Programm Fit2d [7]. Die Distanz Detektor-Probe und die Wellenlänge wurden mittels NIST Standardreferenz 660b LaB_6 bestimmt. Die Probe wurde vor der Messung gemörsert und dann in eine 700 µm Quartz-Kapillare übergeführt. Die Gasphase wurde durch Injektion einer 1:1 Mischung aus Methan und Kohlendioxid (Flussrate 1 mL/min unter Umgebungsbedingungen) bereitgestellt. Die Heizrate unter Trockenreformierbedingungen betrug 20 K/min bis 800 °C.

Details der XPS Auswertung:

Die XP-Daten der Setups 1 und 2 wurden mittels CasaXPS Software (Version 2.3.16 Pre-rel. 1.4) ausgewertet [8]. Ein Shirley Untergrund wurde für die Quantifizierung und das Peak-Fitting benutzt, und die entsprechenden Scofield-Sensitivitätsfaktoren wurden berücksichtigt. Das Peakfitting der $Pd3d_{5/2}$ und C1s-Peaks wurde durch eine gewichtete Summe aus Gauss und Lorentz Peaks mit einem GL(30) Anteil gewährleistet. Asymmetrische Parameter wurden sowohl zum $Pd3d_{5/2}$ Fitten von Pd^0 - GL(30)T(1.3) und Pd_xC - GL(30)T(1.5), sowie zum C1s-Fitten von Graphit - GL(30)T(1.1) und Pd_xC - GL(30)T(1.3) verwendet. Bezüglich der Bindungsenergiezuordnung enthielten alle Pd $3d_{5/2}$ Spektren eine Komponente bei 333.5 eV (angegeben als O1s 2nd harm.), die durch die frequenz-verdoppelte Anregung des O1s Signals (zurückzuführen auf eine Beugung 2. Ordnung der Synchrotron-Strahlung am Gitter des Monochromators) zustande kam. Die Zerlegung aller Pd $3d_{5/2}$ Peaks beinhaltete die Berücksichtigung einer metallischen (bei 335.0 eV) und einer karbidischen Pd_xC Komponente (bei 335.6 eV) [9,10]. Verschiedene C1s-Komponenten wurden in den entsprechenden C1s-Spektren berücksichtigt: Karbonate und Kohlenstoff-Oxygenate oberhalb 287 eV [11] und C_xH_y Spezies bei etwa 285.4 eV [11-13]. Die Komponente bei 283.0 eV wurde interstitiellem/karbidischem C in Pd_xC zugeordnet [14]. Elektronen-Abschwächungslängen wurden aus der NIST-Datenbank SR 82 entnommen [15], die asymmetrischen Orbital-Parameter entsprechend aus der ELETTRA online Datenbank [16].

Zusätzliche Resultate

S1: Schema der zwei benutzten Modellsysteme und deren chemische und strukturelle Veränderung unter Trockenreformierbedingungen. Für den inversen Vorläufer wurde eine simplifizierte Darstellung von fcc (100) Gitterebenen, die Zr^0 Atome in oberflächennahen Bereichen enthalten, gewählt. Dieser Zustand wurde durch XRD und Niederenergieionenstreuung (Setup 1), inklusive des „ZrO_2-Inselzustands" nach oxidativer Segregation unter Reaktionsbedingungen, verifiziert. Die schematische Darstellung des Gitters des volumens-intermetallischen Präkatalysators spiegelt nicht die reale polykristalline Pd_2Zr/Pd_3Zr Phasenzusammensetzung wieder. Unter Reaktionsbedingungen werden Pd^0 und t-ZrO_2 gebildet, welche ebenso nicht mit ihrer realen dreidimensionalen Kristallstruktur abgebildet sind. Beide neu geformten Phasen bilden ein nanokristallines Konglomerat auf dem polykristallinen Pd_xZr_y Substrat. Die Partikelgröße und der Gitterparameter von Pd betragen ~ 7.5 nm und 3.914 Å unter Trockenreformierbedingungen bei 800 °C und ~10.3 nm bzw. 3.898 Å während Oxidation in Sauerstoff.

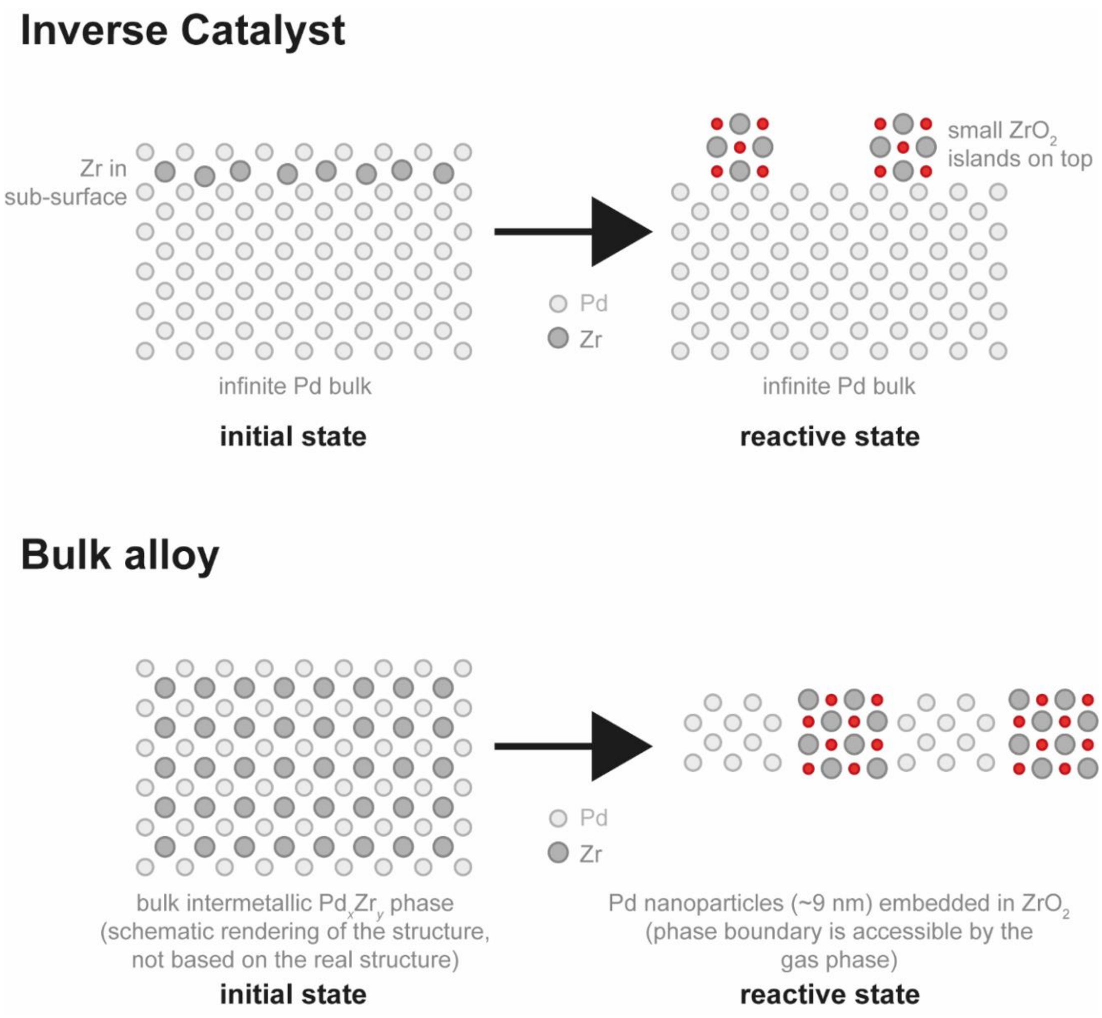

Fig S1: Simplifiziertes Schema der beiden intermetallischen Modellsysteme und deren Transformation unter realistischen Trockenreformierbedingungen.

S2: Oberflächenzustand des volumens-intermetallischen PdZr-Katalysators vor Trockenreformierung. Trotz der Dominanz der Zr^{4+} Spezies in der Zr 3d Region ist die intermetallische Zr^0 Komponente bei einer Bindungsenergie von 179.6 eV [2,3] deutlich sichtbar. Kurzer Kontakt zu Umgebungsbedingungen während des Probentransfers verursachte eine oberflächennahe oxidative Segregation des Bimetalls zu ZrO_2/Pd^0. Die $Pd3d_{5/2}$-Region zeigt ein frequenz-verdoppeltes O1s-Signal bei 333.5 eV, sonst ist ausschließlich metallisches Pd bei 335.0 eV sichtbar. In der C1s-Region ist graphitischer Kohlenstoff schon vor der Trockenreformierung zu sehen, sowie untergeordnete Beiträgen von C_xH_y Spezies und C-Oxygenaten [11,13,17].

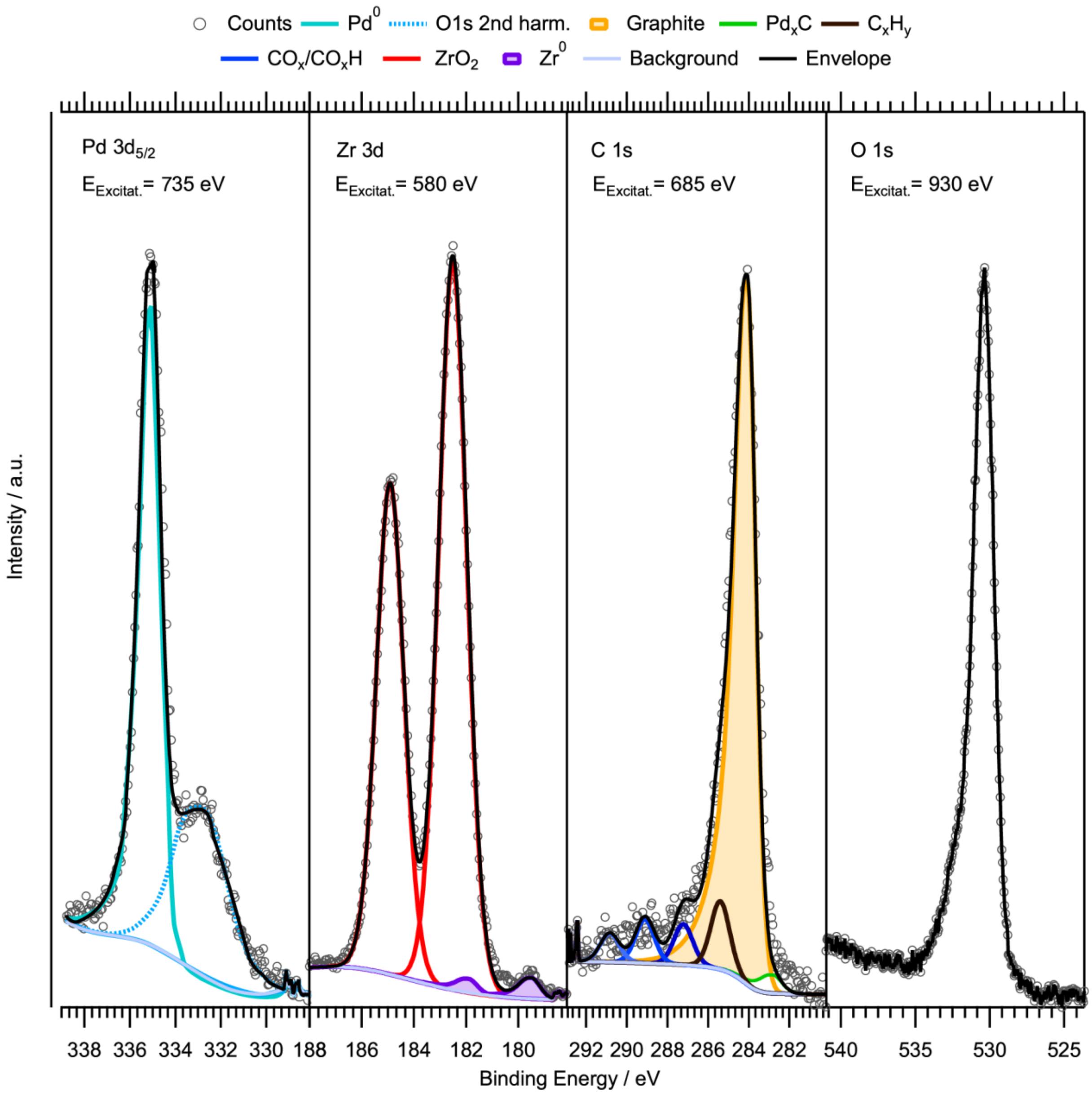

Fig S2: $Pd3d_{5/2}$, Zr3d, C1s und O1s-Regionen des volumens-intermetallischen PdZr-Präkatalysators. Die kinetische Energie der Photoelektronen wurde durch Variation der Photonenenergie für alle Regionen auf 400 eV eingestellt.

S3: Veränderung des Pd-Gitterparameters und der Partikelgröße als Funktion der Reaktionszeit während der Nachoxidation (nach Trockenreformierung) in einem Fluss von 2 ml/min reinem Sauerstoff unter Umgebungsbedingungen und 600 °C, untersucht mit in-situ Röntgenbeugung. Zwischen einer Expositionszeit von 25 und 35 min tritt eine Gitterkontraktion von etwa 0.016 Å ein. Wir nehmen an, dass die Partialoxidation von Pd zu PdO mit einem Verlust von Kohlenstoff im Gitter einhergeht, was wiederum die Gitterkontraktion erklärt.

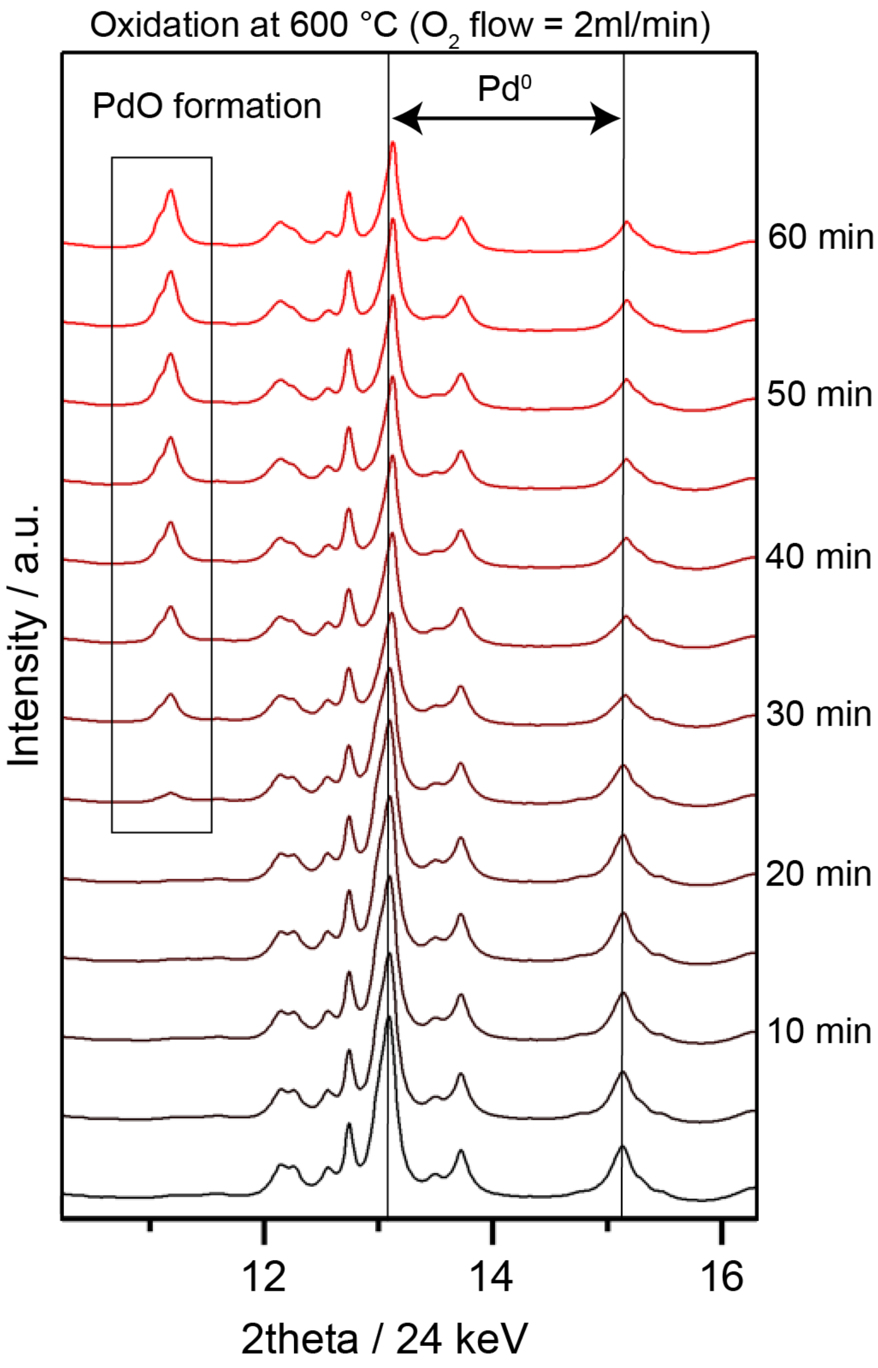

Fig S3: Zeitaufgelöste in-situ XR Diffraktrogramme der Pd/PdO-Region während Nachoxidation in einem Fluss von 2 ml/min reinem Sauerstoff unter Umgebungsdruck und 600 °C. Die Daten wurden aufgenommen, um die Umkehrbarkeit der während der Trockenreformierbedingungen bzw. der Kohlenstoff-Ablagerung auftretenden strukturellen Veränderungen zu testen.

S4: Temperatur-programmierte Trockenreformierprofile auf dem oberflächennahen Intermetallzustand vs. ZrO_2 und reines Pd. Die Reaktionsbedingungen sind ident zu jenen von Abbildung 2 im Haupttext. Nur eine geringfügige Promotierung der Trockenreformieraktivität gegenüber dem reinen ZrO_2-Film wurde beobachtet. Reines Pd is nahezu inaktiv.

Die Präparation von ZrO_xH_y-submonolagenbedecktem Pd^0 via chemischer Gasphasenabscheidung, und in der Folge des „inversen“ oberflächennahen Zr^0-intermetallischen Katalysatorvorläufers auf der Pd Folie, war zu Beginn durch eine potentiell mit der Ausdehnung der Grenzfläche skalierbare Trockenreformieraktivität motiviert. Interessanter Weise ist die beobachtete Aktivität nur unwesentlich größer als die Summe der oxidischen und metallischen Einzelkomponenten. Im Haupttext wird die starke Promotierung der Trockenreformieraktivität beim volumens-intermetallischen Präkatalysator durch die Anwesenheit einer hohen Konzentration von reaktivem gelösten Kohlenstoff erklärt, der via Diffusion durch die metallische Phase an die GF gelangt. Dies ist offensichtlich für den oberflächennahen Intermetall-Zustand auf der Pd-Folie nicht gegeben. Daher verursachen auch Veränderungen des anfänglichen Zr^0-Anteils zwischen 5 und 90% nur kleine Veränderungen in der katalytischen Aktivität. Aus einer Reihe von Gründen sind diese kleinen Unterschiede in der Praxis nicht mit der in-situ (d.h. während der Trockenreformierung) gebildeten Phasengrenze skalierbar.

- Ein schlechtes Signal-zu-Rausch Verhältnis aufgrund der inhärent niedrigen Gesamtaktivität, in Kombination mit zu kleinen Ratenänderungen. Aufgrund der experimentellen Fehlerbalken bei diesen niedrigen Raten kann der relative Anteil der beiden Oberflächenregionen relativ zu den GF-Plätzen nicht quantifiziert werden.

- Nicht skalierbarer Anteile von gelöstem Kohlenstoff im Pd-Volumen (Konzentration senkrecht zur Oberfläche ist Zeit-, Temperatur-, und Oxidbedeckungs-abhängig)

- Nicht skalierbare Inseldichte von resegregiertem ZrO_xH_y aus dem bimetallischen Vorläufer, d.h. die genaue Anzahl der GF-Plätze ist nicht bekannt. Gleichzeitig ist die genaue nanoskopische Verteilung dieser Inseln nicht bestimmbar, d.h. die Raten können nicht darauf normalisiert werden. In-situ-Mikroskopie unter Trockenreformierbedingungen wäre notwendig um dies zu untersuchen, ist aber experimentell außer Reichweite.

- Selbst bei erfolgreicher Bestimmung der Verteilung dieser Inseln wäre eine skalierbare Relation zwischen GF-Plätzen und Aktivität aufgrund der oben diskutierten Gründe wiederum nicht möglich.

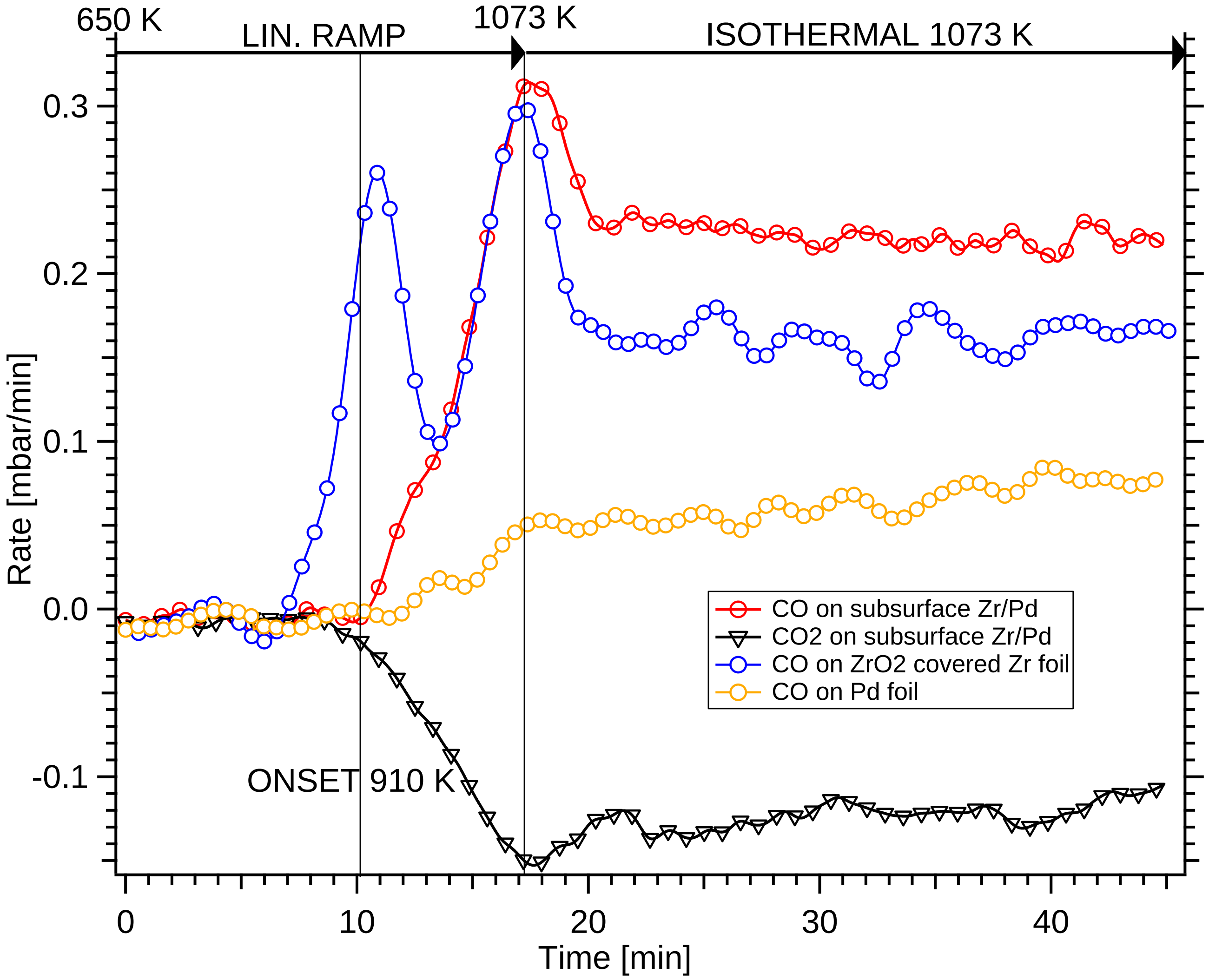

Fig S4: Temperatur-programmierte Trockenreformier-Aktivität gemessen am ursprünglichen oberflächennahen Pd^0Zr^0 Intermetall-Zustand in einer 1:1 Mischung aus Methan und Kohlendioxid (je 50 mbar). Heizrate 25 K/min, isotherme Reaktion für 30 min bei 800°C.

S5: Relative Intensitätsvariationen von C_{bulk}, $C_{Graphit}$ und $C_{oxygenat}$ C1s-Intensitäten mit der XPS-Informationstiefe. Die Oxygenat-Intensität zeigt einen relativ zu $C_{Graphit}$ offensichtlich erhöhten Signalanteil bei hohen Photonenenergien. Es ist klar, dass eine volumengelöste Spezies wie C_{bulk} eine reduzierte Signalabschwächung mit Erhöhung der Photonenenergie/ der Probentiefe/ der kinetischen Photoelektronenenergie aufweisen sollte, speziell in Relation zu den oberflächengebundenen Graphen/Graphit-Lagen. In Analogie dazu erscheint die C1s-Intensität der Oxygenate weniger abgeschwächt als die von $C_{Graphit}$. Wie in Schema 1 gezeigt, erzeugt die Korrosion der volumens-intermetallischen PdZr-Probe ein Konglomerat aus Pd^0-Nanopartikeln und Domänen aus t-ZrO_2, das automatisch einen hohen Anteil aus „vergrabenen" GF-Plätzen enthält. Eine erhöhte Konzentration von Oxygenatspezies an diesen Plätzen würde einer quasi-3D Verteilung normal zur äußeren (geometrischen) Oberfläche entsprechen und deshalb eine zu C_{bulk} vergleichbare Intensitätsantwort auf die Photonenenergievariation liefern. Wir betrachten dies derzeit allerdings als Spekulation, da die schlechte Datenqualität und die Probleme der Untergrundkorrektur der C1s-Region im Bereich der Oxygenat-Spezies keine verlässliche Zuordung von Intensitätstrends zulässt.

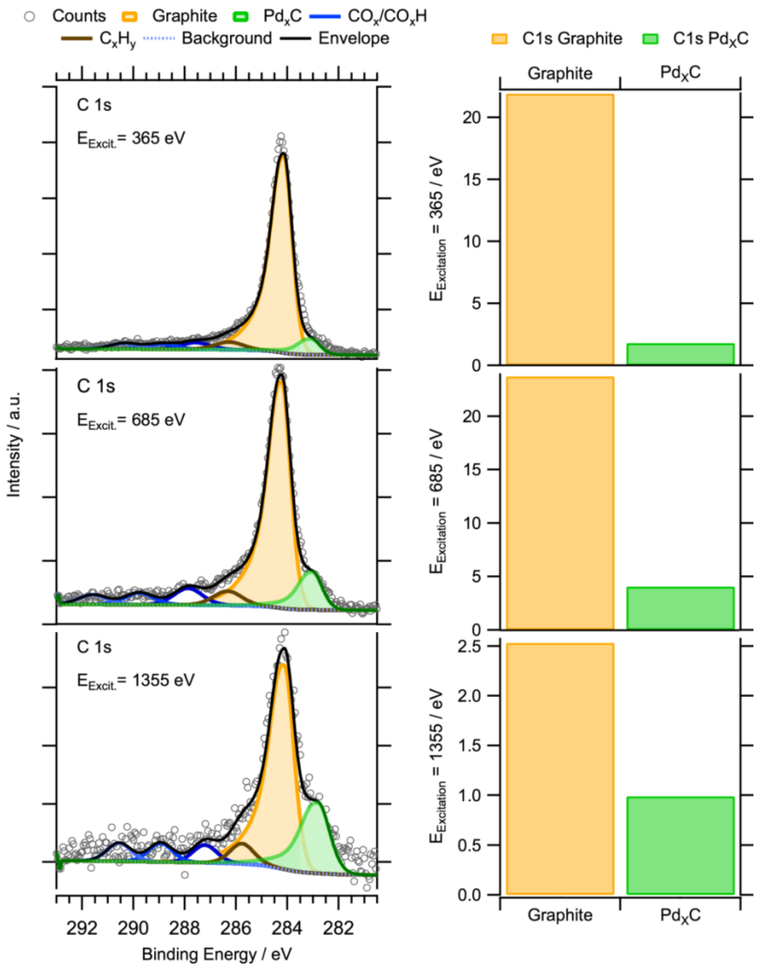

Fig S5: In-situ Tiefenprofil des C1s-Signals unter Trockenreformierbedingungen unter konstanten Druckbedingungen. T=700 °C, Partialdrücke von Methan und Kohlendioxid jeweils 0.15 mbar.

S6: Vergleich der anfänglichen und in-situ beobachteten Kohlenstoffsignale in reinem Kohlendioxid auf dem anfänglichen volumens-intermetallischen PdZr-Katalysator. In reinem Methan wurde der Kohlenstoff-Konzentrationslevel im linken Spektrum von Abbildung 3A im Haupttext erzeugt, danach wurde die Probe 150 min lang in 0.3 mbar reinem Kohlendioxid bei ~740 °C spektroskopiert. Wir merken an, dass sich die Kohlenstoff-Abreaktion bei einem Konzentrationslevel knapp unterhalb des anfänglichen merklich verlangsamt, und sehr viel langsamer von statten geht als die Abreaktion des Trockenreformier-induzierten $C_{Graphit}$-Anteils. Dies bedeutet, dass der zurückbleibende C-Anteil, der auf den Kontakt mit den Umgebungsbedingungen beim Transfer zurückzuführen ist, für die CO_2-Reaktion kinetisch weniger zugänglich ist. Nach 150 min musste das Experiment aufgrund limitierter Strahlzeit abgebrochen werden. Über eine Standardquantifizierung unter der Annahme homogen verteilter Elemente konnten aus den Peakflächen von Abbildung S6 Werte von 11.3 Atom.-% C auf der anfänglichen und 8.5 Atom.-% C auf der in-situ CO_2-behandelten Probe ermittelt werden. Über die chemische Natur, das Diffusionsverhalten oder die Verteilung dieser Kohlenstoff-Spezies kann nur spekuliert werden und ebenso können für die Verweilzeit/Stabilität in reinem Kohlendioxid nur grobe Abschätzungen vorgenommen werden. Auf der Basis einer Abnahme von etwa 2-3 % innerhalb von 2-3 Stunden würde es ca. 6 Stunden dauern, um den größten Anteil abzureagieren. Eine mögliche Erklärung wäre, dass dieser „atmosphäreninduzierte“ Kohlenstoff weiter von der GF entfernt gebunden ist und/oder ein in anderer Weise behindertes Diffusionsverhalten aufweist.

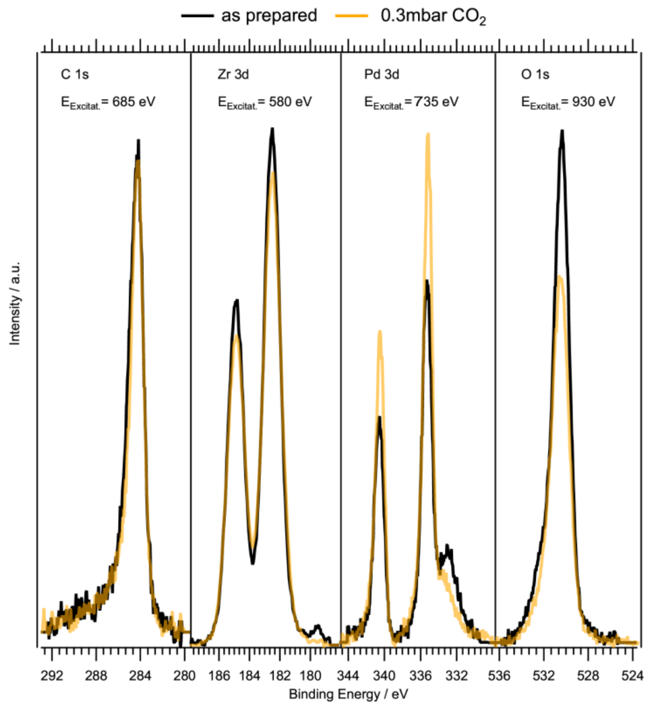

Fig S6**:** Relative Intensitäten der C1s-, Zr3d-, Pd3d- und O1s-Regionen, bestimmt bei gleicher kinetischer Energie der Photoelektronen von 400 eV. Schwarz: anfänglicher Zustand vor Katalyse, dunkelgelb: während Abreaktion in 0.3 mbar Kohlendioxid nach 150 min bei 740 °C. Beide Spektrensets wurden bei demselben Gesamtdruck aufgenommen, um vergleichbare Gasphasenabsorptionseigenschaften zu ermöglichen.

S7: In-situ XP-Spektren auf dem ursprünglichen oberflächennahen Intermetallzustand unter Trockenreformierbedingungen. Die gasphaseninduzierten Intensitätstrends sind rechts im Säulendiagramm gezeigt. Eine totale Kohlenstoff-Abreaktion wurde in reinem Kohlendioxid beobachtet. Der Kohlenstoff, der nach dem Wachstum in reinem Methan erscheint, befindet sich bevorzugt auf den ZrO_2 Inseln, die in-situ gebildet werden. Im Vergleich zu reinem Methan wird unter Trockenreformierbedingungen eine geringere und zeitunabhängige stationäre Kohlenstoff-Konzentration beobachtet.

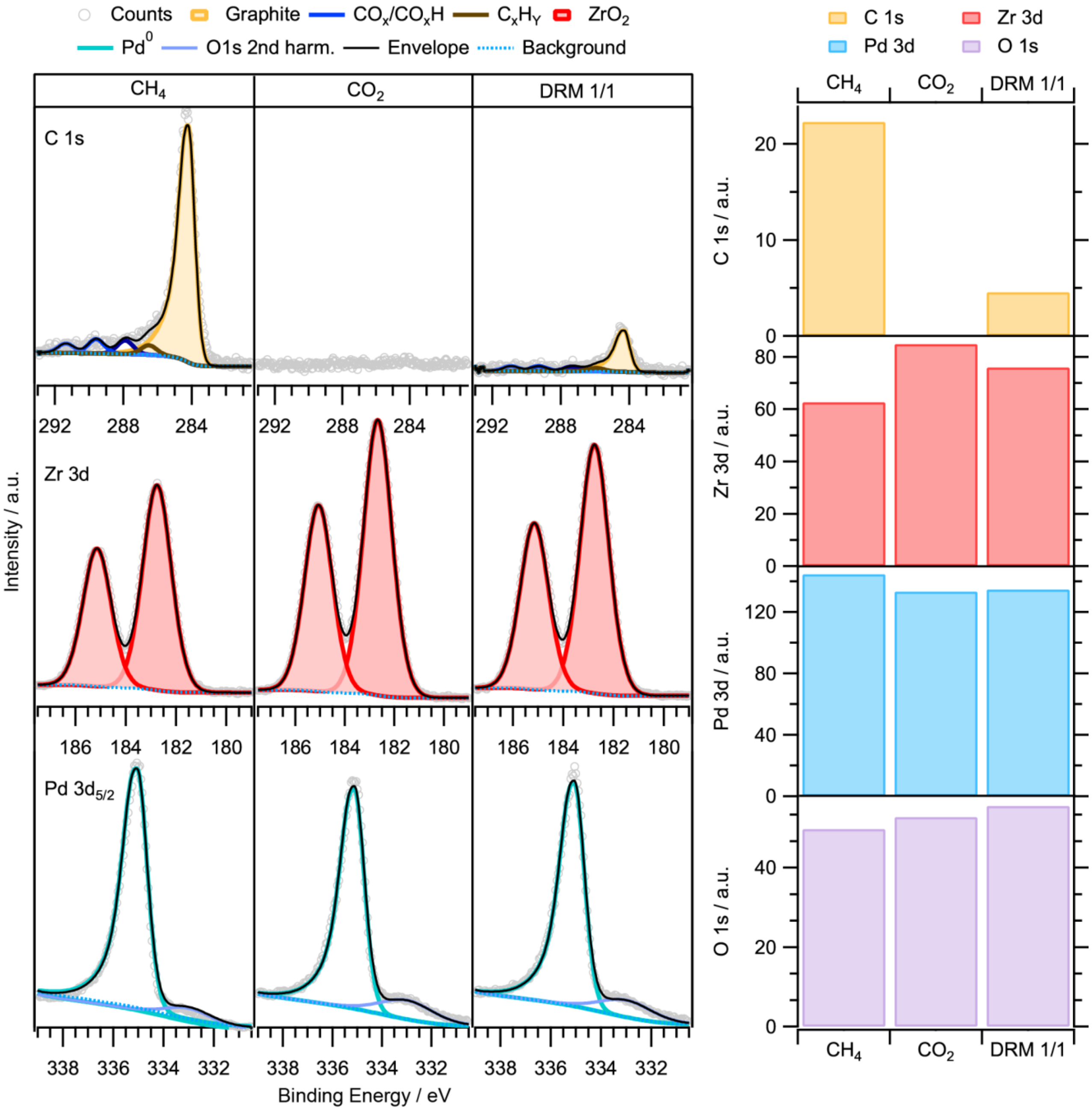

Fig S7: C1s-, Zr3d- und Pd3d-XP-Spektren, in-situ gemessen mit einer gemeinsamen kinetischen Energie von 400 eV bei ~ 700 °C am anfänglichen oberflächennahen PdZr-Intermetallzustand. Links: 0.3 mbar reines Methan, Mitte: 0.3 mbar reines Kohlendioxid, rechts: Trockenreformiermischung 0.15 mbar Methan und 0.15 mbar Kohlendioxid. Ganz rechts: Balkendiagramm der Intensitätstrends.

S8: In-situ-Variation der stationären C_{bulk}- und $C_{graphit}$-Intensitäten als Funktion der Temperatur unter Trockenreformierbedingungen. Zwischen 600 und 700 °C steigen die Intensitäten beider Komponenten, oberhalb von 700 °C nehmen beide ab, wobei die relative Reaktion der C_{bulk}-Komponente stärker erscheint. Bis 700°C könnte dies auf eine Erhöhung der Kohlenstoff-Löslichkeit zurückzuführen sein, sowie auf die gleichzeitig verstärkte Methan-Zersetzungsrate mit Erhöhung der Temperatur – d.h. die Pd-Partikel weisen also eine erhöhte Netto-Kohlenstoff-Sättigung auf. Oberhalb von 700 °C ist die Kohlenstoff-Abreicherung thermodynamisch begünstigt, da ΔG der Methanzersetzung mit Erhöhung der Temperatur abnimmt, allerdings langsamer als ΔG der inversen Boudouard Reaktion. Die Temperatur, bei der die Rate der Oxidation des Kohlenstoffs durch Kohlendioxid die der Kohlenstoff-Abscheidung überholt, liegt bei etwa 725°C, wie bereits in [18] berichtet wurde. Ein entsprechendes mechanistisches Modell, das bei Temperaturerhöhung eine beschleunigte Abreicherung von C_{bulk} durch Reaktion zu CO via GF berücksichtigt, könnte diese Trends erklären. Eine detaillierte Zuordnung der Abreicherungsrate von C_{bulk} und seiner Nachlieferung, sowohl via Wiederauflösung von Graphit und durch kontinuierliche Kohlenstoff – Nachlieferung via Methan, wurde bis dato nicht versucht.

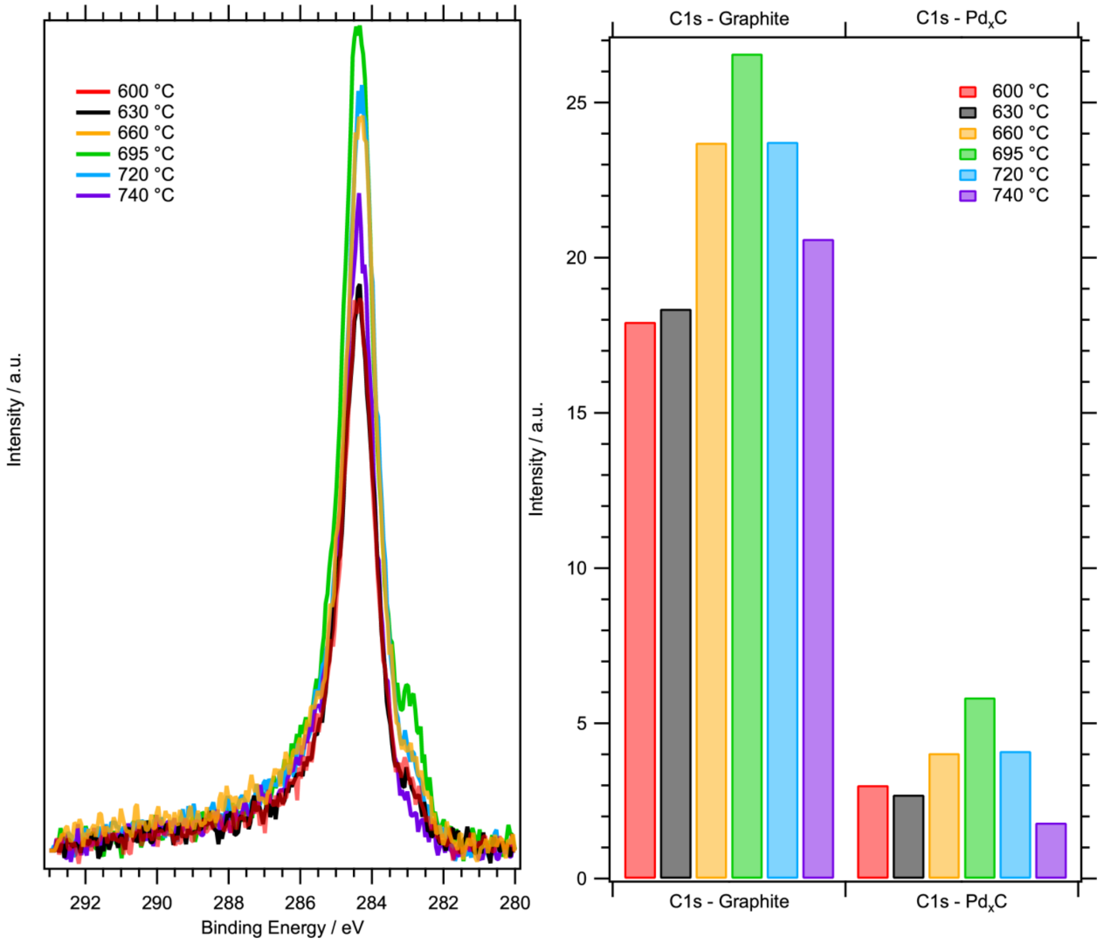

Fig. S8: Antwort der stationären C_{bulk} und $C_{Graphit}$-Intensitäten auf Temperaturvariation zwischen 600°C und 740 °C. Konstante Druckverhältnisse während der Trockenreformierung: jeweils 0.15 mbar Methan und Kohlendioxid. Kinetische Energie der Photoelektronen 400 eV, hν=685 eV.

Literatur zur Zusatzinformation:

[1] L. Mayr, X.-R. Shi, N. Köpfle, C. A. Milligan, D. Y. Zemlyanov, A. Knop-Gericke, M. Hävecker, B. Klötzer, S. Penner, *Phys. Chem. Chem. Phys.* **2016**, *18*, 31586.

[2] N. Köpfle, L. Mayr, P. Lackner, M. Schmid, D. Schmidmair, T. Götsch, S. Penner, B. Kloetzer, *ECS Trans.* **2017**, *78*, 2419.

[3] H. Li, J.-I. J. Choi, W. Mayr-Schmölzer, C. Weilach, C. Rameshan, F. Mittendorfer, J. Redinger, M. Schmid, G. Rupprechter, *J. Phys. Chem. C* **2015**, *119*, 2462.

[4] L. Mayr, R. Rameshan, B. Klötzer, S. Penner, C. Rameshan, *Rev. Sci. Instrum.* **2014**, *85*, 55104.

[5] H. Bluhm, M. Hävecker, A. Knop-Gericke, M. Kiskinova, R. Schlögl, M. Salmeron, *MRS Bull.* **2007**, *32*, 1022.

[6] a) L. Schlicker, A. Doran, P. Schneppmüller, A. Gili, M. Czasny, S. Penner, A. Gurlo, *Rev. Sci. Instrum.* **2018**, *89*, 33904; b) A. Doran, L. Schlicker, C. M. Beavers, S. Bhat, M. F. Bekheet, A. Gurlo, *Rev. Sci. Instrum.* **2017**, *88*, 13903.

[7] A. P. Hammersley, S. O. Svensson, M. Hanfland, A. N. Fitch, D. Hausermann, *High Pressure Res.* **1996**, *14*, 235.

[8] *CasaXPS Version 2.3.16 Pre-rel 1.4*, Casa Software Ltd, **2011**.

[9] O. Balmes, A. Resta, D. Wermeille, R. Felici, M. E. Messing, K. Deppert, Z. Liu, M. E. Grass, H. Bluhm, R. van Rijn et al., *Phys. Chem. Chem. Phys.* **2012**, *14*, 4796.

[10] D. Teschner, J. Borsodi, A. Wootsch, Z. Révay, M. Hävecker, A. Knop-Gericke, S. D. Jackson, R. Schlögl, *Science* **2008**, *320*, 86.

[11] A. Wolfbeisser, B. Klötzer, L. Mayr, R. Rameshan, D. Zemlyanov, J. Bernardi, K. Föttinger, G. Rupprechter, *Catal. Sci. Technol.* **2015**, *5*, 967.

[12] N.M. Rodriguez, P.E. Anderson, A. Wootsch, U. Wild, R. Schlögl, Z. Paál, *J. Catal.* **2001**, *197*, 365.

[13] D. N. Belton, S. J. Schmieg, *J. Vac. Sci. Technol., A* **1990**, *8*, 2353.

[14] B. S. Ahn, S. G. Jeon, H. Lee, K. Y. Park, Y. G. Shul, *Appl. Catal., A* **2000**, *193*, 87.

[15] C. J. Powell and A. Jablonski, *NIST Electron Effective-Attenuation-Length Database SRD 82,*, Instititue of Standards and Technology, Gaithersburg, **2011**.

[16] J. J. Yeh, *Atomic Calculation of Photoionization Cross-Sections and Asymmetry Parameters*, Gordon and Breach Science Publishers, Langhorne, PE, USA, **1993**.

[17] a) Z. Bastl, *Collect. Czech. Chem. Commun.* **1995**, *60*, 383; b) G.J. Kovács, I. Bertóti, G. Radnóczi, *Thin Solid Films* **2008**, *516*, 7942; c) N. Laidani, L. Calliari, G. Speranza, V. Micheli, E. Galvanetto, *Surf. Coat. Technol.* **1998**, *100-101*, 116.

[18] M. C. J. Bradford, M. A. Vannice, *Catal. Rev. Sci. Eng.* **1999**, *41*, 1.
